# Direct targets of *MEF2C* are enriched for genes associated with schizophrenia and cognitive function and are involved in neuron development and mitochondrial function

**DOI:** 10.1371/journal.pgen.1011093

**Published:** 2024-09-11

**Authors:** Deema Ali, Aodán Laighneach, Emma Corley, Saahithh Redddi Patlola, Rebecca Mahoney, Laurena Holleran, Declan P. McKernan, John P. Kelly, Aiden P. Corvin, Brian Hallahan, Colm McDonald, Gary Donohoe, Derek W. Morris

**Affiliations:** 1 Centre for Neuroimaging, Cognition and Genomics (NICOG), University of Galway, Ireland; 2 School of Biological and Chemical Sciences, University of Galway, Ireland; 3 School of Psychology, University of Galway, Ireland; 4 Discipline of Pharmacology & Therapeutics, School of Medicine, University of Galway, Ireland; 5 Neuropsychiatric Genetics Research Group, Department of Psychiatry, Trinity College Dublin, Ireland; 6 Discipline of Psychiatry, School of Medicine, University of Galway, Ireland; Duke-NUS: Duke-NUS Medical School, SINGAPORE

## Abstract

*Myocyte Enhancer Factor 2C* (*MEF2C*) is a transcription factor that plays a crucial role in neurogenesis and synapse development. Genetic studies have identified *MEF2C* as a gene that influences cognition and risk for neuropsychiatric disorders, including autism spectrum disorder (ASD) and schizophrenia (SCZ). Here, we investigated the involvement of *MEF2C* in these phenotypes using human-derived neural stem cells (NSCs) and glutamatergic induced neurons (iNs), which represented early and late neurodevelopmental stages. For these cellular models, *MEF2C* function had previously been disrupted, either by direct or indirect mutation, and gene expression assayed using RNA-seq. We integrated these RNA-seq data with *MEF2C* ChIP-seq data to identify dysregulated direct target genes of *MEF2C* in the NSCs and iNs models. Several *MEF2C* direct target gene-sets were enriched for SNP-based heritability for intelligence, educational attainment and SCZ, as well as being enriched for genes containing rare *de novo* mutations reported in ASD and/or developmental disorders. These gene-sets are enriched in both excitatory and inhibitory neurons in the prenatal and adult brain and are involved in a wide range of biological processes including neuron generation, differentiation and development, as well as mitochondrial function and energy production. We observed a trans expression quantitative trait locus (eQTL) effect of a single SNP at *MEF2C* (rs6893807, which is associated with IQ) on the expression of a target gene, *BNIP3L*. *BNIP3L* is a prioritized risk gene from the largest genome-wide association study of SCZ and has a function in mitophagy in mitochondria. Overall, our analysis reveals that either direct or indirect disruption of *MEF2C* dysregulates sets of genes that contain multiple alleles associated with SCZ risk and cognitive function and implicates neuron development and mitochondrial function in the etiology of these phenotypes.

## Author summary

Schizophrenia is a complex disorder caused by many genes. Current drugs for schizophrenia are only partially effective and do not treat cognitive deficits, which are key factors for explaining disability, leading to unemployment, homelessness and social isolation. Large-scale genetic studies of schizophrenia and cognitive function have been effective at identifying individual SNPs and genes that contribute to these phenotypes but have struggled to immediately uncover the bigger picture of the underlying biology of the disorder. Here we take an individual gene associated with schizophrenia and cognitive function called *MEF2C*, which on its own is a very important regulator of brain development. We use functional genomics data from studies where *MEF2C* has been mutated to identify sets of other genes that are influenced by *MEF2C* in developing and mature neurons. We show that several of these gene-sets are enriched for common variants associated with schizophrenia and cognitive function, and for rare variants that increase risk of various neurodevelopmental disorders. These gene-sets are involved in neuron development and mitochondrial function, providing evidence that these biological processes may be important in the context of the molecular mechanisms that underpin schizophrenia and cognitive function.

## Introduction

*MEF2C*, a transcription factor within the myocyte enhancer factor-2 (MEF2) family, is involved in essential neurodevelopmental processes [1]. *MEF2C* is expressed during the initial stages of embryonic brain development and remains expressed at elevated levels in adult brains, including in the striatum, hippocampus, and cortex, indicating an involvement in both embryonic and adult brain activity [1,2]. *MEF2C* plays a critical role in neurogenesis, neuronal distribution and electrical activity in the neocortex [3–5]. Mutations in the *MEF2C* gene, including microdeletions, missense, or nonsense mutations, have been linked to a rare genetic disorder known as *MEF2C* haploinsufficiency syndrome. This syndrome is characterized by intellectual disability (ID), epilepsy, and additional autistic features like absent speech and impaired social interactions [6]. Genome-wide association studies (GWAS) have identified common variants in the *MEF2C* gene that are associated with schizophrenia (SCZ) intelligence (IQ) and educational attainment (EA) [7–9]. *MEF2C* is associated with genetic and epigenetic risk architectures of SCZ [10]. *MEF2C* motifs were present among the top-scoring single-nucleotide polymorphisms (SNPs) associated with SCZ in GWAS and deep sequencing of histone methylation landscapes in individuals with SCZ and controls revealed a significant abundance of *MEF2C* motifs associated with histone hypermethylation in the disorder. Additionally, the upregulation of *MEF2C* improved working memory, object recognition memory, and spinal remodeling in prefrontal projection neurons in mice [10].

Various studies have utilized *MEF2C* heterozygous or homozygous knockout (KO) animal models to investigate the role of *MEF2C* in brain function and to identify molecular mechanisms underlying human phenotypes associated with *MEF2C* [3,11–15]. Recently, Mohajeri *et al*. (2022) evaluated *MEF2C* loss-of-function mutations in human-derived cell lineages representing different stages of neural development [16]. They directly disrupted the gene by targeting the coding sequence of *MEF2C* with CRISPR-engineered mutations, resulting in 122kb and 131kb deletions of the gene. Expanding beyond direct disruption of the gene, they utilized an indirect approach to disrupt the 3D genome organization of the locus and the regulatory architecture of the gene. Here, they either deleted the distal boundary (DB) or the proximal boundary (PB) of the topologically associated domain (TAD) encompassing *MEF2C*. Specifically, they performed a targeted deletion of a 3.3kb segment of the DB, which targeted a single occupied CTCF binding site located more than 1.3Mb distal to the *MEF2C* promoter. As for the PB, they carried out a targeted deletion of a single occupied CTCF binding site within a 3’ intron of *MEF2C*. Following the direct or indirect mutation of *MEF2C* in human induced pluripotent stem cells (iPSCs), these cells were differentiated into neural stem cells (NSCs) and glutamatergic induced neurons (iNs) as cellular models [16]. NSCs are undifferentiated cells that have the ability to self-renew and generate various types of neurons and glial cells. Glutamatergic iNs are responsible for synthesizing glutamate, the primary excitatory neurotransmitter in the mammalian central nervous system. Glutamate plays a crucial role in various essential brain processes, including cognition, learning, memory, and sensory perception [17]. The study used these cellular models to investigate the impact of both direct and indirect disruptions of *MEF2C* on global transcriptional signatures and electrophysiological changes in human neurons [16]. Both the direct disruption and the loss of a PB, but not the deletion of a DB, led to down-regulation of *MEF2C* expression, which resulted in reduced synaptic activity. The presence of common differentially expressed genes (DEGs) associated with neurogenesis and neuronal differentiation in both direct and indirect *MEF2C* disruptions suggests shared functional consequences arising from both types of *MEF2C* disruption [16].

**Fig 1:**
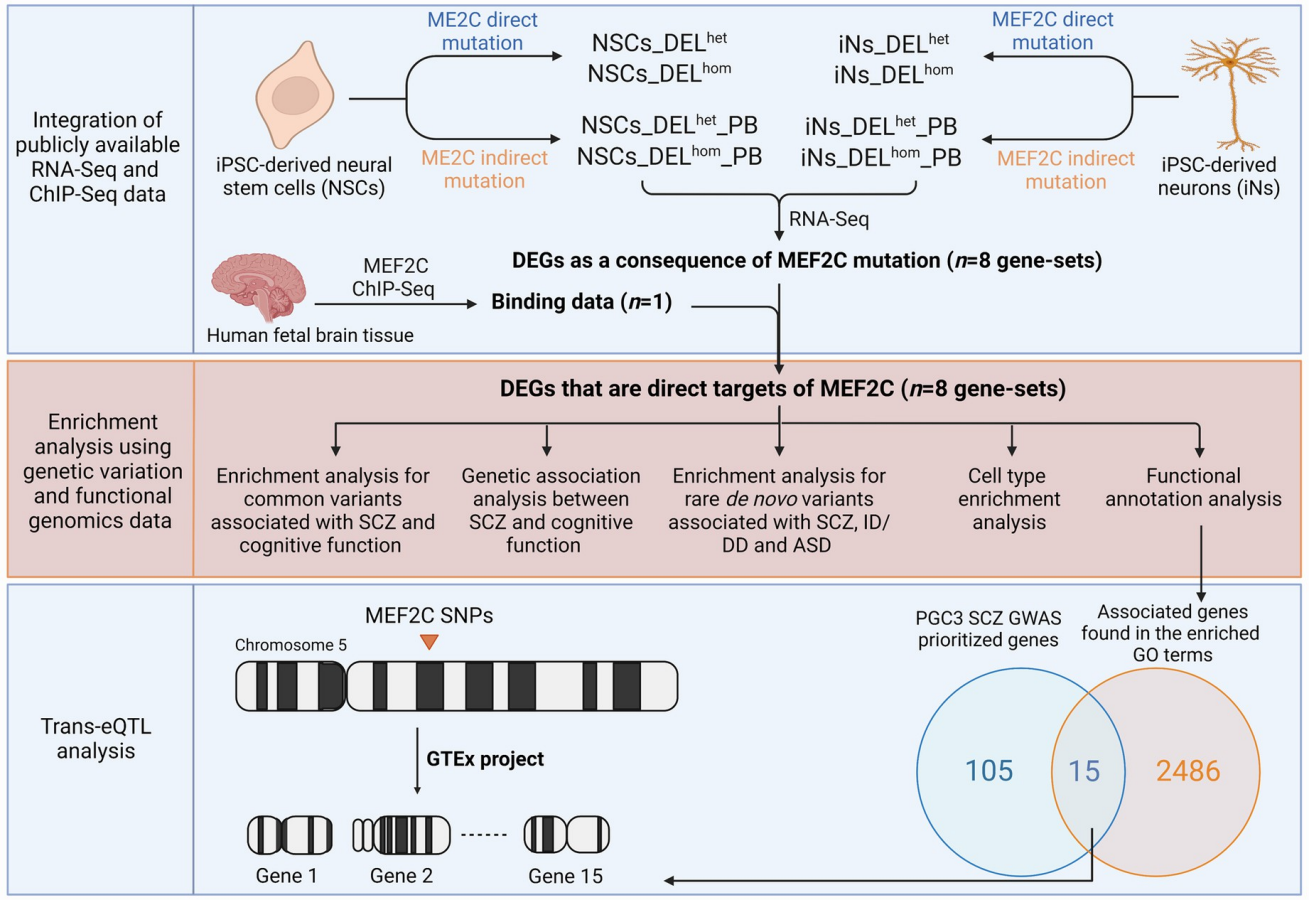
Schematic representation illustrating the stepwise methodology employed in this study. DELhom: Homozygous deletion; DELhet: Heterozygous deletion; PB: Proximal boundary (indirect mutation of MEF2C); DEGs: Differentially expressed genes; SCZ: Schizophrenia; ID: Intellectual disability; DD: Developmental delay; ASD: Autism spectrum disorder; GO: Gene ontology; PGC: Psychiatric Genomics Consortium; GWAS: Genome-wide association study; SNPs: Single nucleotide polymorphisms; eQTL: Expression quantitative trait loci; GTEx: Genotype-Tissue Expression. Figure created with BioRender.com.

Here, we expanded upon the findings of Mohajeri *et al*. (2022) [16] by utilizing their gene expression data and combining it with chromatin immunoprecipitation sequencing (ChIP-seq) data for *MEF2C* (Fig 1) [18]. This integration enabled us to identify putative direct transcriptional targets of *MEF2C* in both NSCs and iNs that were dysregulated following either heterozygous or homozygous direct or indirect mutation of the gene. Given *MEF2C*’s association with neuropsychiatric disorders and cognitive function, we sought to investigate if the direct targets of *MEF2C* that are dysregulated by different mutations in the different cellular models are enriched for genes containing SNPs associated with SCZ and cognitive function from GWAS, as well as enriched for genes harboring rare *de novo* mutations (DNMs) contributing to neurodevelopmental disorders. Subsequently, we investigated the biological processes and specific cell types that are dysregulated as a consequence of *MEF2C* disruption to better understand the contribution of *MEF2C*-regulated genes to the molecular mechanisms of SCZ and cognition. Finally, we sought to identify trans-expression quantitative trait loci (trans-eQTL) at the *MEF2C* gene that are associated with altered expression of downstream *MEF2C* target genes (Fig 1).

## Materials and methods

### Ethics statement

Data were directly downloaded from published studies and no additional ethics approval was needed. Each study is referenced and details on ethics approval are available in each manuscript.

### *MEF2C* Transcriptomic data

We utilized transcriptomic data generated in a study of *MEF2C* conducted by Mohajeri *et al*. (2022) [16]. That study used dual-guide CRISPR-Cas9 genome editing to directly (deletion in the coding region) or indirectly (mutation of the PB of the TAD that encompasses *MEF2C*) disrupt *MEF2C* function in human iPSCs. Single cells were isolated and screened to identify edited clones and matched unedited controls. Six replicates per genotype (heterozygous (het) or homozygous (hom) deletion (DEL)) were then differentiated into NSCs and iNs and subjected to RNA-seq analysis. This analysis generated eight sets of differentially expressed genes (DEGs) in these cellular models, labelled as follows with PB denoting the indirect mutation: NSCs_DEL^het^, NSCs_DEL^hom^, NSCs_DEL^het^_PB, NSCs_DEL^hom^_PB, iNs_DEL^het^, iNs_DEL^hom^, iNs_DEL^het^_PB, and iNs_DEL^hom^_PB. Each gene-set represented a different combination of *MEF2C* disruption and genotype in NSCs or iNs. The significant DEGs were identified at FDR < 0.1 (S1 Table).

### *MEF2C* ChIP-sequencing data analysis

To investigate *MEF2C* binding, we utilized existing ChIP-seq data from a study of *MEF2C* conducted on human fetal brain cultures [18]. Input DNA was used as a control. The raw files, comprising a single replicate, were provided by the authors. The quality of the raw FastQ files was assessed using the FastQC (http://bioinformatics.babraham.ac.uk/projects/fastqc/). Reads were aligned to the human genome (hg19) using Burrows Wheeler Aligner (BWA; http://bowtie-bio.sourceforge.net/bowtie2) [19]. Post-processing of the alignment data was conducted using Samtools (https://github.com/samtools). We converted the SAM files to BAM format, sorted the BAM files, removed any potential PCR duplicates and generated a file containing mapping statistics [20]. Peaks were called using MACS2 (parameters: -f BAM -g hg -q 0.01) [21]. ChIPSeeker was used to determine overlap with genomic features and for peak annotation to the nearest genes [22].

### Integrative Analysis of RNA-Seq and ChIP Data

To infer the direct target genes of *MEF2C*, the eight sets of DEGs described above were integrated with the *MEF2C* ChIP-seq data using the BETA (Binding and Expression Target Analysis) (http://cistrome.org/BETA/) package software [23]. BETA ranks genes based on two key factors: the regulatory potential of factor binding sites and the differential expression observed upon factor binding. The regulatory potential is assessed by considering the distance of the binding sites from the transcription start site and the cumulative impact of multiple binding sites. By considering both aspects, a rank product (RP) was calculated for each gene, which can be interpreted as a probability, indicating the likelihood that a gene is a true direct target of *MEF2C* based on both criteria. Genes with a conservative RP < 0.01 were considered as direct targets of *MEF2C* (S2 Table).

### Stratified linkage disequilibrium score regression (sLDSC) Analysis

Stratified linkage disequilibrium score regression (sLDSC) (https://github.com/bulik/ldsc) [24] was used to investigate if the *MEF2C* direct targets were enriched for heritability contributing to SCZ, IQ, and EA phenotypes. GWAS summary statistics for these phenotypes [7–9] were obtained from publicly available databases (the Psychiatric Genomics Consortium Website www.med.unc.edu/pgc, the Complex Trait Genetics lab www.ctg.cncr.nl/, and the Social Science Genetic Association Consortium www.thessgac.org/data). For control purposes, we performed sLDSC analysis using GWAS summary statistics for an additional four phenotypes, including attention deficit hyperactivity disorder (ADHD) [25], obsessive–compulsive disorder (OCD)[26], Alzheimer’s disease (AD) [27] and stroke [28]. Linkage disequilibrium (LD) scores between SNPs were estimated using the 1000 Genomes Phase 3 European reference panel. SNPs present in HapMap 3 with an allele frequency > 0.05 were included. Enrichment of heritability was assessed controlling for the effects of 53 functional annotations included in the full baseline model version. Enrichment for heritability was compared to the baseline model using the Z-score to derive a (one-tailed) P-value. Significant enrichments were determined using a Bonferroni correction, which set the corrected P value threshold at < 2.08E-03.

### Overlapping Genes Implicated in the GWAS of SCZ and IQ/EA

In the GWAS of IQ, 1,016 genes were reported as associated with IQ through positional mapping, eQTL mapping, chromatin interaction mapping and gene-based association analysis [8]. For EA, 1,838 genes were prioritized using Data-driven Expression *Prioritized* Integration for Complex Traits (DEPICT), which was based on correlations across reconstituted gene-sets [9]. For SCZ, 682 associated genes were identified through fine mapping and summary-data-based mendelian randomization [7] (S3 Table). To investigate distinct and overlapping associations with SCZ and cognitive function, we identified genes that are associated with SCZ but not IQ or EA (n = 472), genes that are associated with at least one of the cognitive phenotypes (IQ or EA) but not SCZ (n = 2,258) and genes that are associated with both SCZ and at least one of the cognitive phenotypes (IQ or EA; n = 210).

### Gene-set based polygenic risk score (PRS)

PRSice-2 software [20] was utilized for gene-set based PRS analysis aiming to investigate whether the *MEF2C* target gene-sets contributed to the shared genetic basis of SCZ and cognitive traits. PRSice-2 calculates PRS for each individual by summing up the number of minor alleles at each SNP multiplied by the GWAS effect size. It performs regression analysis, adjusting for sex, age, and GWAS array type as covariates, and provides performance metrics (Nagelkerke’s R2 and P value). SNP P values and effect sizes for SCZ were derived from a SCZ GWAS meta-analysis on 40,675 cases and 64,643 controls [7]. Irish samples were excluded from this GWAS to keep that base/discovery sample independent from the target/test sample for the PRS analysis. The SNP P values and effect sizes for IQs were based on an IQ GWAS on 269,867 individuals [8]. For each gene-set, SNPs in high LD were clumped according to PRSice-2 guidelines. Genotype data for the identified SNPs were extracted from the full GWAS data of the Irish samples, which consisted of 1,512 individuals, including SCZ patients and controls with IQ measurements [29,30]. Effect-size weighted SCZ-PRS and IQ-PRS were computed for each gene-set using thresholds ranging from P < 0.05 to P≤1 (*P* < *0*.*05*, 0.1, 0.15, 0.2, *1*). To validate the findings, 10,000 randomized phenotypes (equally distributed cases and controls as per the original dataset) were generated from the Irish samples, and SCZ-PRS and IQ-PRS were calculated for each gene-set using the randomized data to obtain empirical P values.

### Analysis of *De Novo* Mutations

The R package denovolyzeR (http://denovolyzer.org/) was used to test for enrichment of rare *de novo* mutations (DNMs) in our gene-sets, estimating the expected number of DNMs for each gene based on sequence context and gene size [31]. Synonymous (Syn), missense (Mis), and loss of function (Lof) (including nonsense, frameshift, and splice) DNMs reported in exome sequencing studies of SCZ (*n* = 3447 trios) [32–35], ASD (*n* = 6430 trios) [36], and ID and/or DD (*n* = 4,851 trios) [37–40] and unaffected siblings (*n* = 1,995) [32]. S4 Table provides details about each study along with the respective table names listing the identified DNMs. To ensure consistency with the Deciphering Developmental Disorders Study (2017), we applied a filtering step for DNMs. Specifically, DNMs with a posterior probability score below 0.00781 were excluded. Enrichment of DNMs in a gene-set was investigated using a two-sample Poisson rate ratio test, using the ratio of observed to expected DNMs in genes outside of the gene-set as a background model. Significant enrichments were determined using a Bonferroni correction, which set the corrected P value threshold at < 5.21E-04.

### *MEF2C* Direct Target Genes Analysis with Single-cell RNA-seq

The Expression Weighted Cell-type Enrichment (EWCE) R package (https://github.com/NathanSkene/EWCE) was used to assess if the direct target genes of *MEF2C* had higher expression in a particular cell type than expected by chance [41]. This method generates random gene sets (*n* = 100,000) of equal length from background genes to estimate the probability distribution. We performed enrichment analysis in a prenatal human dataset and in an adult human dataset. The prenatal human dataset included single-nuclei RNA sequencing (snRNA-seq) data from three fetuses from the second trimester of gestation and contained data for 91 distinct clusters of nuclei from five brain regions (frontal cortex (FC), ganglionic eminence (GE), hippocampus (Hipp), thalamus (Thal), and cerebellum (Cer)) [42]. The adult human dataset included data for 120 distinct clusters of nuclei from across the human cortex covering the middle temporal gyrus (MTG), cingulate gyrus (CgG), primary visual cortex (V1C), primary somatosensory cortex (S1C) and the primary motor cortex (M1C). Nuclei were sampled from postmortem and neurosurgical (MTG only) donor brains (https://portal.brain-map.org/atlases-and-data/rnaseq/protocols-human-cortex) [43,44]. The significance of the enriched expression of the *MEF2C* direct target genes relative to the background genes in each cell type was assessed by calculating the difference in standard deviations between the two expression profiles. Statistically significant enrichments were determined using a Bonferroni correction to adjust for multiple testing of cell types.

### Functional enrichment analysis

ClueGO (version 2.5.9), a plugin for Cytoscape (version 3.8.2) was used to identify the Gene Ontology (GO) terms (categorized as biological processes (BP), molecular functions (MF), and cellular components (CC)) and the biological pathways (KEGG, Reactome, WikiPathways) enriched for (i) genes proximal to MEF2C peaks identified via ChIP-seq analysis using brain tissue-expressed genes as the background gene-set (S5 Table) [45] and (ii) the *MEF2C* direct target gene-sets using specific cell-type expressed genes as the background gene-set. Brain tissue-expressed genes were obtained directly from the Human Protein Atlas database (https://www.proteinatlas.org/) [46]. Cell type-specific expressed genes were identified by calculating Transcripts Per Million (TPM) values from raw reads counts of wild type NSCs and iNs downloaded from the gene expression omnibus (GEO GSE204778). Genes with TPM values less than 1 were filtered out. GO term relationships were determined based on shared genes and assessed using chance-corrected kappa statistics. Bonferroni correction was applied to adjust for multiple testing.

## Results

### Identification and Annotation of MEF2C Binding Peaks

Analysis of ChIP-Seq data using MACS and ChIPSeeker identified 10,620 MEF2C binding peak regions (FDR ≤ 1%) across the entire genome. Approximately 80% of the peaks were located in close proximity to gene-encoding regions including promoters (< = 1-kb (55.8%), 1–2 kb (2.35%), 2–3 kb (1.99%)), 5’ UTRs and 3’ UTRs (0.44%), exons (0.18%), first introns (5.8%), and other intron regions (14.8%) (S6 Table). When the binding peaks were mapped to the closest RefSeq annotated transcripts, they were found in close proximity to 5,775 protein coding genes. GO annotation analysis showed these genes were most involved in RNA binding, transcription and functions within the nucleus (S7 Table).

### Identification of *MEF2C* Direct Target Genes

We integrated *MEF2C* ChIP-seq data with data on DEGs from cell line models where *MEF2C* had been mutated (2 cell types (NSCs or iNs) x 2 DEL mutation types (direct or indirect (PB)) x 2 genotypes (het or hom) = 8 cell line models). Fig 1 illustrates the stepwise methodology employed in this study, from generating the eight gene-sets from these models through to enrichment analysis using genetic variation and functional genomics data. We identified that *MEF2C* had a direct regulatory influence on approximately 23–57% of the DEGs from the original study (Table 1). Shared *MEF2C* direct target genes were observed between different genotypes within the same cell type in both NSCs and iNs. In NSCs, the proportion of shared genes for both heterozygous and homozygous genotypes ranged from 37% (direct mutation) to 42% (indirect mutation), while in iNs, it ranged from 23% (direct mutation) to 34% (indirect mutation) (S1 Fig). Furthermore, there was a limited number of common *MEF2C* direct target genes found for the same genotype across different gene disruption types, ranging from 13% (homozygous) to 22% (heterozygous) in NSCs and from 8% (homozygous) to 18% (heterozygous) in iNs (S1 Fig). A small fraction of the *MEF2C* direct target genes (3% in NSCs and 1.4% in iNs) were in each gene-set (S1 Fig), indicating that the downstream effect of different gene mutations and their genotypic state is to mostly impact distinct sets of genes.

**Table 1:** Number of DEGs in each gene-set before and after the integration with MEF2C ChIP-seq data to identify direct target genes.

Gene-Set	# of all DEGs	# of *MEF2C* Direct Targets (% of MEF2C Direct Targets relative to all DEGs)
**NSCs**		
DEL^het^	366	169 (46%)
DEL^hom^	2187	1170 (53%)
DEL^het^_PB	492	240 (49%)
DEL^hom^ _PB	728	412 (57%)
**iNs**		
DEL^het^	689	335 (51%)
DEL^hom^	291	145 (50%)
DEL^het^_PB	2980	1034 (35%)
DEL^hom^ _PB	5132	1174 (23%)

NSCs: Neural stem cells; iNs: Induced neurons; DEL^hom^: Homozygous deletion; DEL^het^: Heterozygous deletion; PB: Proximal boundary (indirect mutation of MEF2C).

### Enrichment analysis for genes containing common variants

**Fig 2:**
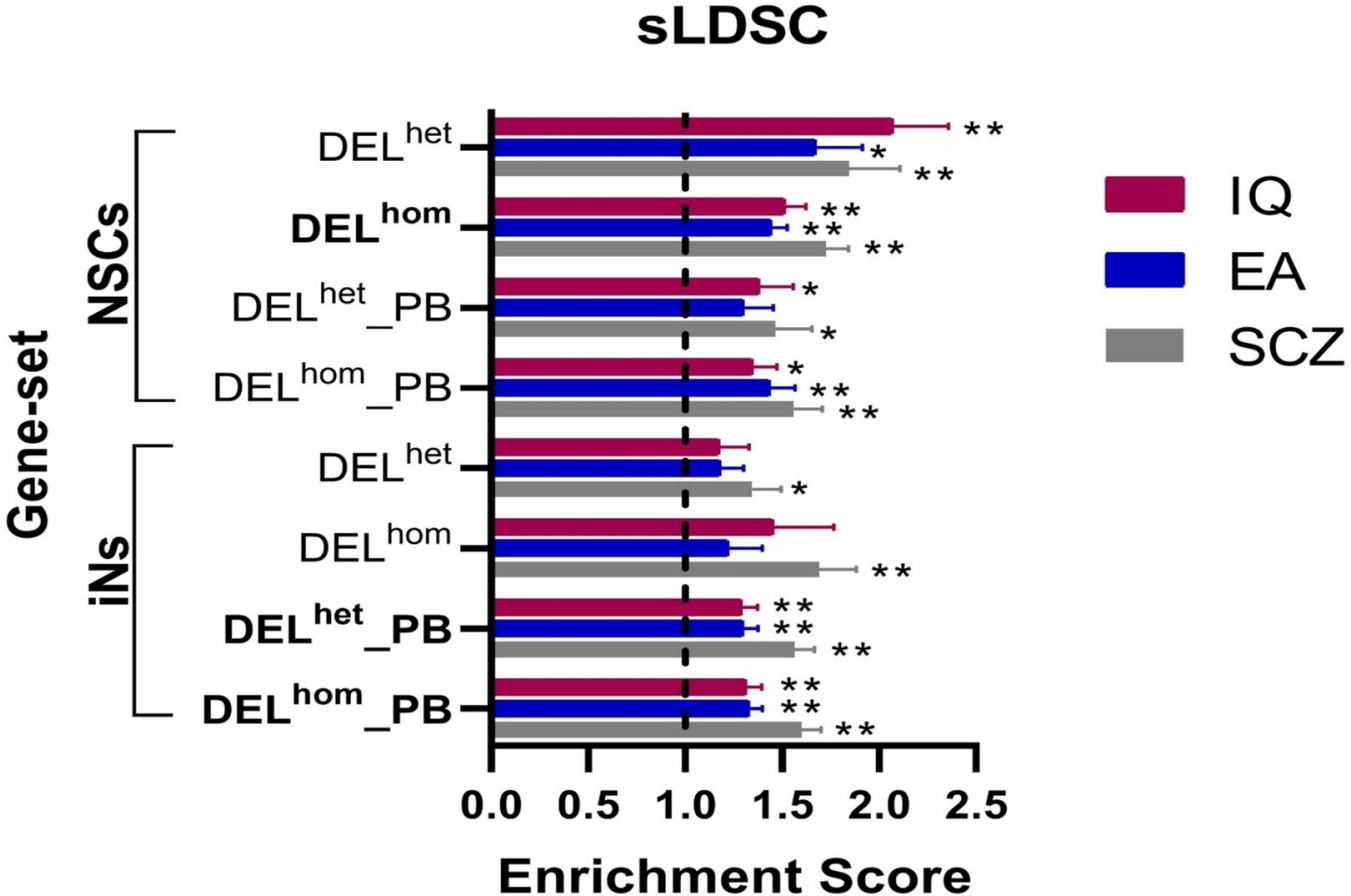
Results from sLDSC analysis of MEF2C direct target gene-sets using GWAS data. The graph plots the enrichment values, defined as the ratio of heritability (h2) to the number SNPs, on the x-axis. The error bars represent the standard errors. Two asterisks (**) indicate significance after Bonferroni correction, and one asterisk (*) indicates nominal significance. Gene-sets enriched for the three phenotypes are highlighted in bold. NSCs: Neural stem cells; iNs: induced neurons; DELhom: Homozygous deletion; DELhet: Heterozygous deletion; PB: Proximal Boundary.

We performed sLDSC analysis to investigate if the *MEF2C* direct target gene-sets are enriched for genes containing common genetic variants associated with SCZ risk or cognitive ability [7–9]. Six of the eight gene-sets were significantly enriched for heritability contributing to at least one of the studied phenotypes (SCZ, IQ, and/or EA) (Fig 2). Specifically, three gene-sets (NSCs_DEL^hom^, iNs_DEL^het^_PB, and iNs_DEL^hom^_PB) were significantly enriched for all phenotypes after multiple testing correction (Fig 2 and S8 Table). These findings highlight the potential role of *MEF2C* in regulating genes involved in SCZ and cognitive function. When we removed genes associated with SCZ from the enrichment analysis of IQ and EA, we saw that the majority of enriched gene-sets remained significant. When we removed genes associated with IQ or EA from the enrichment analysis of SCZ, we saw that only the enrichment of the NSCs_DEL^hom^ gene-set remained significant (S9 Table). This suggests that some *MEF2C* target genes are contributing to both SCZ and cognitive phenotypes while others are more phenotype specific. No significant enrichment was detected for any of the four control phenotypes (S10 Table).

### Genetic Association between SCZ and cognitive function

**Fig 3:**
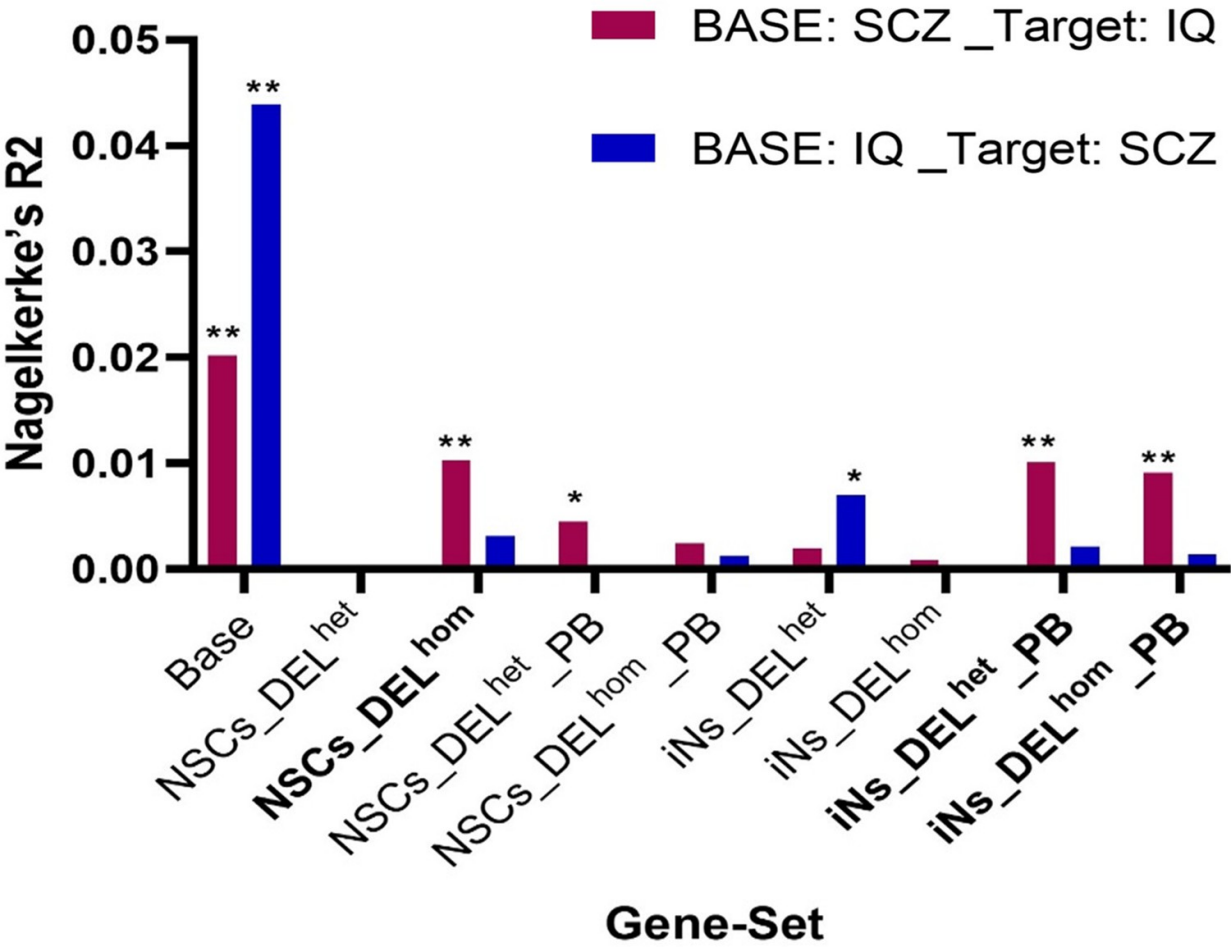
Gene-set based PRS analysis to examine associations between the SCZ-PRS and IQ, and between the IQ-PRS and SCZ. Base on the x-axis refers to a PRS generated using all variants in the genome. The height of the columns on the y-axis indicates the proportion of variance in the phenotype explained when a gene-set based PRS is constructed using SCZ GWAS data and is tested against IQ (pink columns) or when a gene-set based PRS is constructed using IQ GWAS data and is tested against SCZ case-control status (blue columns). Two asterisks (**) indicate significance after Bonferroni correction, and one asterisk (*) indicates nominal significance. NSCs: Neural stem cells; iNs: induced neurons; DELhom: Homozygous deletion; DELhet: Heterozygous deletion; PB: Proximal Boundary.

To explore the genetic overlap between these phenotypes further, gene-set based PRS analysis was conducted to investigate if the *MEF2C* target gene-sets contributed to the shared genetic etiology between SCZ and cognition. This was done by generating a gene-set PRS based on SCZ risk from GWAS and testing if this SCZ-PRS could explain variance in IQ in an independent dataset. We also tested if a gene-set IQ-PRS could predict SCZ case-control status in independent dataset. While the IQ-PRS could not predict SCZ case-control status, we found that the SCZ-PRS derived from three of the eight gene-sets (NSCs_DEL^hom^, iNs_DEL^het^_PB, and iNs_DEL^hom^_PB) could explain a significant proportion of variance in IQ (Fig 3 and S11 Table). These are the same three gene-sets that were previously enriched for common variation associated with SCZ, IQ and EA. When we removed genes associated with IQ or EA from these gene-sets, these three SCZ-PRSs could still explain variance in IQ in an independent dataset at levels that were nominally significant (S12 Table). These findings suggest that genetic variants associated with SCZ within these gene-sets also influence cognitive performance and the effect was not just due to genes already associated with IQ or EA. We performed a sensitivity analysis with respect to the p-value threshold for SNP inclusion and found that results were consistent and stable across different p-value thresholds (S11 and S12 Tables).

### Enrichment Analysis for Genes Containing *De Novo* Mutations

To assess the impact of rare variants within the *MEF2C* gene-sets on SCZ and other neurodevelopmental disorders where cognitive impairment is a major feature (ASD, ID and DD), we examined whether these gene-sets exhibited enrichment for Syn, Mis, and Lof DNMs in trio-based exome sequencing studies of these disorders [32–40]. The same gene-sets that showed enrichment for common variants associated with SZ, IQ and EA (NSCs_DEL^hom^, iNs_DEL^het^_PB, and iNs_DEL^hom^_PB) were also significantly enriched for genes containing Lof and/or Mis DNMs reported specifically in ID and/or DD patients after multiple test correction (Table 2). The NSCs_DEL^hom^ gene-set was also significantly enriched for Lof DNMs found in people with autism (Tables 2 and S13). None of our gene-sets showed enrichment for genes containing rare DNMs reported in SCZ patients. As a control, our gene-sets were not enriched for Syn DNMs reported for these disorders and not enriched for any class of DNM reported in the unaffected siblings of patients.

**Table 2:** Rare variant enrichment analysis of MEF2C direct target gene-sets using data on DNMs, identified in patients with SCZ, ASD, ID and DD.

Gene-Set	SCZ *n* = 3394 trios	ASD *n* = 6430 trios	ID/DD *n* = 4485 trios	Unaffected Siblings *n* = 1995
**NSCs**				
DEL^het^	ns	ns	ns	ns
DEL^hom^	ns	Lof**	Mis**, Lof**	ns
DEL^het^_PB	ns	Lof*	ns	ns
DEL^hom^ _PB	ns	Lof*	Mis	ns
**iNs**				
DEL^het^	ns	ns	Mis*	ns
DEL^hom^	Syn*	ns	Mis*, Lof*	ns
DEL^het^_PB	ns	ns	Mis**	ns
DEL^hom^ _PB	Lof*	Mis*, Lof*	Mis**, Lof**	ns

Two asterisks (**) indicate significant enrichment for mutation type after Bonferroni correction, one asterisk (*) indicates significant enrichment for mutation type at nominal significance level and ns indicates non-significant for all classes of mutation tested. SCZ: Schizophrenia; ASD: Autism spectrum disorder; ID: Intellectual disability; DD: Developmental delay; Syn: Synonymous mutations, Mis: Missense mutations; Lof: Loss-of-function mutations; NSCs: Neural stem cells; iNs: Induced neurons; DEL^hom^: Homozygous deletion; DEL^het:^ Heterozygous deletion; PB: Proximal boundary (indirect mutation of MEF2C).

### Cell-type enrichment analysis

We utilized the EWCE R package [41] to investigate which individual cell types are enriched for these genes in the prenatal and adult human brain using snRNA-seq data. The three gene-sets with by far the most enriched cell types are the three gene-sets that were enriched for common variation associated with SCZ, IQ and EA and rare DNMs reported in neurodevelopmental disorders (NSCs_DEL^hom^, iNs_DEL^het^_PB and iNs_DEL^hom^_PB). There is a consistent pattern for these gene-sets in the prenatal and adult data with both glutamatergic excitatory neurons and GABAergic inhibitory neurons enriched across different regions of the prenatal brain and across regions of the adult cortex (S14 and S15 Tables). The enrichment of genes in both excitatory and inhibitory neurons is consistent with the role of *MEF2C* in regulating the balance of excitatory and inhibitory synapses, the disruption of which may contribute to neurodevelopmental disease [11]. Lastly, the NSCs_DEL^hom^ gene-set was enriched within cycling progenitor cells and intermediate progenitor cells within the prenatal brain, which like the NSCs can produce new types of neurons and glial cells (S14 Table).

### Functional enrichment analysis

**Fig 4:**
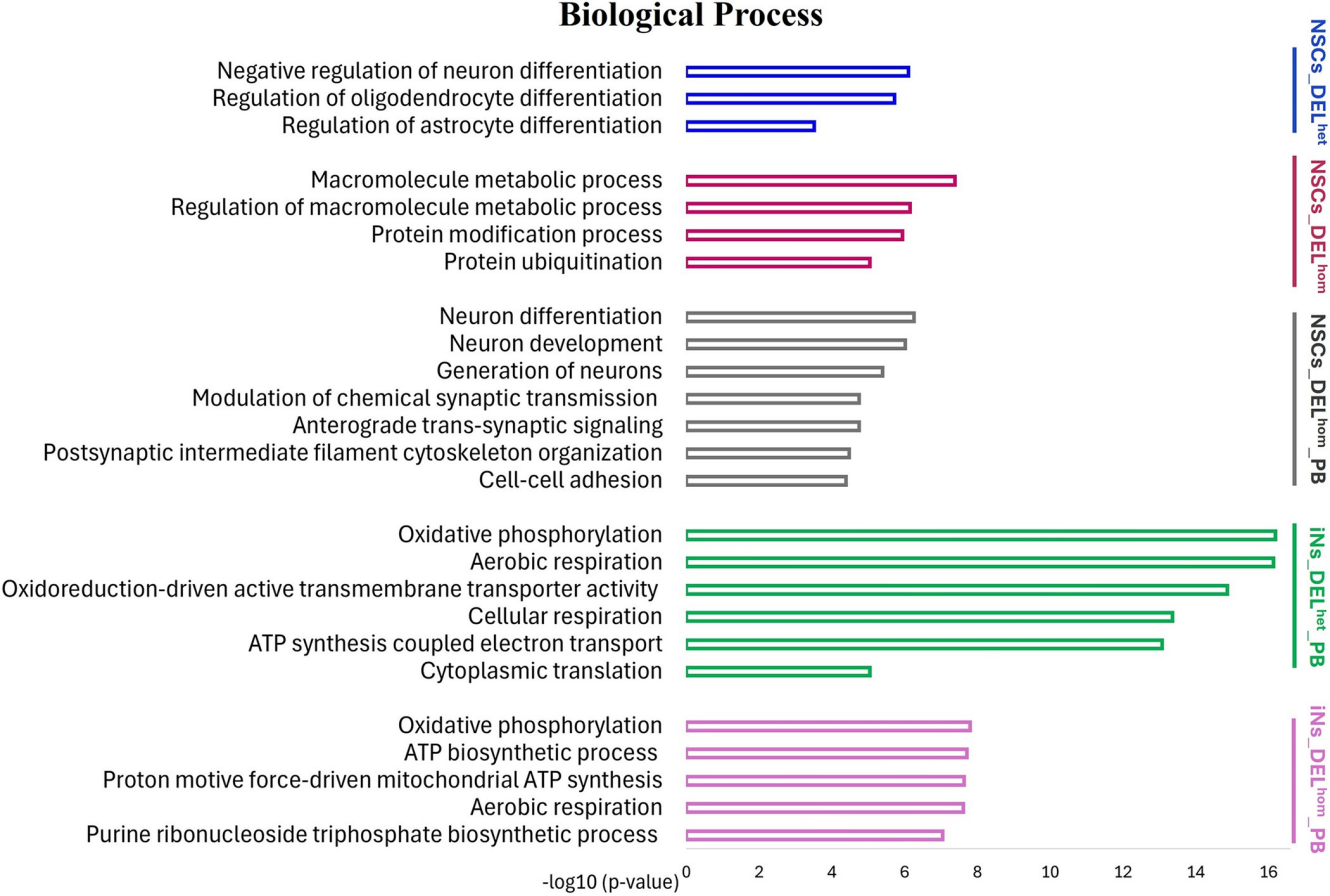
Bar charts of gene ontology (GO) analysis of biological process for MEF2C direct target gene-sets using the ClueGo plugins of Cytoscape. The Bonferroni method was applied for a p-value correlation (p < 0.05). The vertical axis displays the names of the GO terms. The horizontal axis and bar lengths represent the significance [−log10 (p-value)]. Colors in the bars represent different MEF2C direct target gene-sets. Results are presented only for the five gene-sets that were previously enriched for common variation associated with SCZ, IQ and/or EA. Enriched terms that were related to each other in the ontology were grouped together, with the most significant term(s)/group displayed. All data is detailed in S16–S23 Tables. NSCs: Neural stem cells; iNs: induced neurons; DELhom: Homozygous deletion; DELhet: Heterozygous deletion; PB: Proximal Boundary.

**Fig 5:**
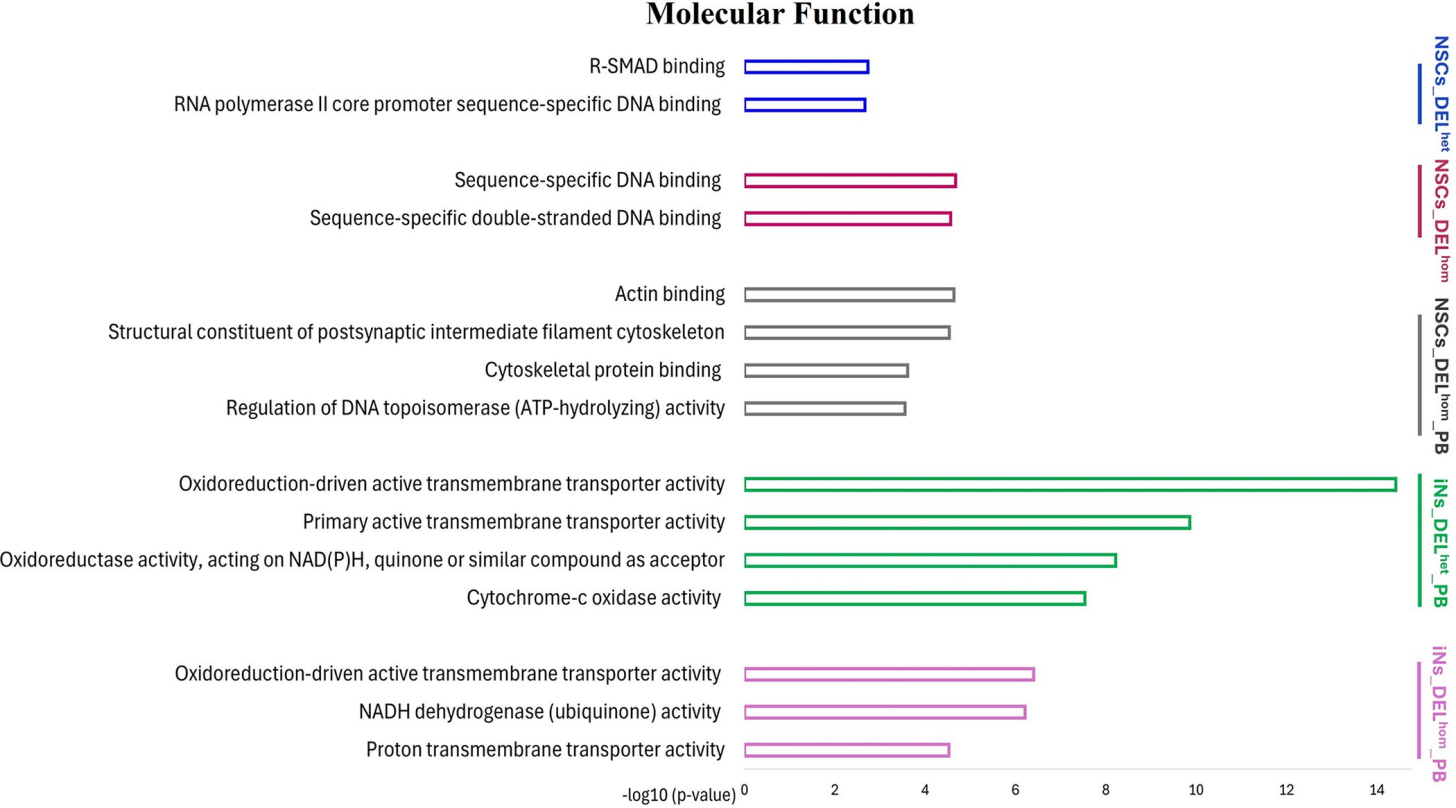
Bar charts of gene ontology (GO) analysis of molecular function for MEF2C direct target gene-sets using the ClueGo plugins of Cytoscape. The Bonferroni method was applied for a p-value correlation (p < 0.05). The vertical axis displays the names of the GO terms. The horizontal axis and bar lengths represent the significance [−log10 (p-value)]. Colors in the bars represent different MEF2C direct target gene-sets. Results are presented only for the five gene-sets that were previously enriched for common variation associated with SCZ, IQ and/or EA. Enriched terms that were related to each other in the ontology were grouped together, with the most significant term(s)/group displayed. All data is detailed in S16–S23 Tables. NSCs: Neural stem cells; iNs: induced neurons; DELhom: Homozygous deletion; DELhet: Heterozygous deletion; PB: Proximal Boundary.

**Fig 6:**
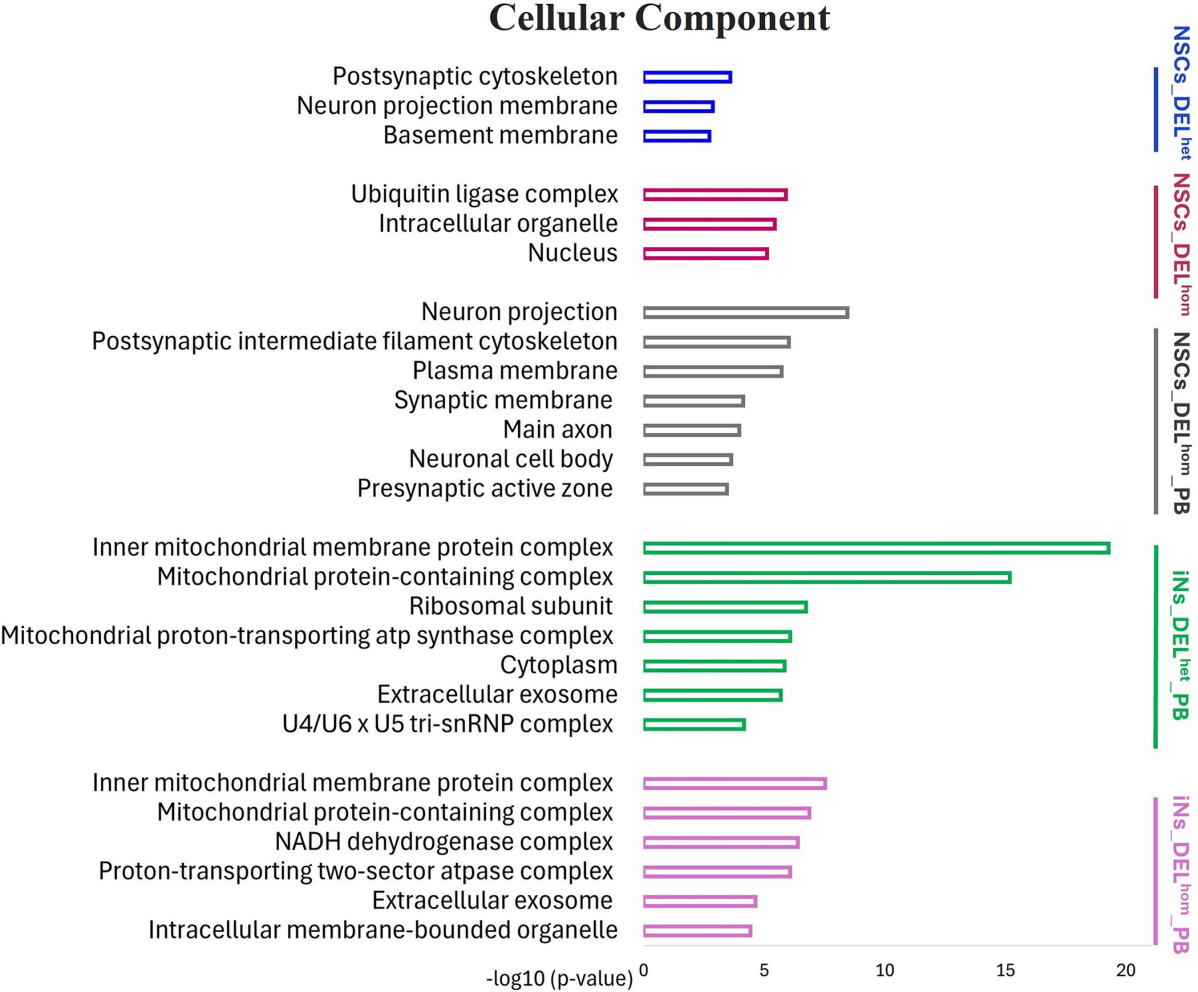
Bar charts of gene ontology (GO) analysis of cellular component for MEF2C direct target gene-sets using the ClueGo plugins of Cytoscape. The Bonferroni method was applied for a p-value correlation (p < 0.05). The vertical axis displays the names of the GO terms. The horizontal axis and bar lengths represent the significance [−log10 (p-value)]. Colors in the bars represent different MEF2C direct target gene-sets. Results are presented only for the five gene-sets that were previously enriched for common variation associated with SCZ, IQ and/or EA. Enriched terms that were related to each other in the ontology were grouped together, with the most significant term(s)/group displayed. All data is detailed in S16–S23 Tables. NSCs: Neural stem cells; iNs: induced neurons; DELhom: Homozygous deletion; DELhet: Heterozygous deletion; PB: Proximal Boundary.

ClueGO (version 2.5.9), a plugin for Cytoscape (version 3.8.2) was used to investigate if genes within the eight sets are over-represented in similar or distinct GO terms for biological processes, cellular components and molecular functions, and biological pathways, using specific cell-type expressed genes as the background gene-set. The NSCs gene-sets were enriched for GO terms related to neuron development, regulation of neuron and glial cell differentiation and regulation of metabolic processes (Figs 4–6 and S16–S19 Tables). The iNs gene-sets were enriched for GO terms related to mitochondrial function and energy production, including the oxidative phosphorylation process (Figs 4–6 and S20–S23 Tables). KEGG pathway analysis revealed that the NSCs gene-sets were enriched in pathways including *Orexin receptor pathway*, *Protein processing in endoplasmic reticulum and Aerobic glycolysis*, while the iNs gene-sets were enriched in pathways including *The citric acid (TCA) cycle and respiratory electron transport* and *Oxidative phosphorylation* (S16–S23 Tables). The enriched GO terms following disruption of MEF2C are distinct from those observed earlier from the ChIP-seq binding pattern of MEF2C in the absence of any gene disruption.

### Trans expression quantitative trait loci analysis

We hypothesized that genetic variation at *MEF2C* (associated with SCZ, IQ or EA) could indirectly affect expression of a downstream target gene, mediated through MEF2C’s role as a transcription factor. This would be a trans expression quantitative trait loci (eQTL) effect and evidence of two risk genes (i.e., *MEF2C* and a downstream target gene) functioning within a putative risk pathway. To reduce the number of possible tests of target genes, we first restricted this analysis to the five gene-sets that were enriched for association with at least one of SCZ, IQ or EA. We next limited these *MEF2C* direct target genes to only those in significantly enriched GO terms (to capture genes with relevant functions; S24 Table) and to those genes among the 120 genes prioritized in the latest GWAS for SCZ (S25 Table) [7]. These 120 genes were identified through a combination of fine-mapping, transcriptomic analysis and functional genomic annotations [7]. The GWASs of IQ and EA had not performed similar prioritization analysis and each reported >1,000 associated genes. Fifteen of the 120 genes are *MEF2C* direct target genes that were in the enriched GO terms (S26 Table). We took 10 LD-independent SNPs at *MEF2C* that were associated with SCZ, EA, and IQ at genome-wide significant levels (S27 Table) and investigated their association with the expression levels of these fifteen genes using eQTL data obtained from the Genotype-Tissue Expression (GTEx) project (https://gtexportal.org/home/) [47]. We detected a trans eQTL for a single SNP at *MEF2C* (rs6893807; associated with IQ in GWAS) on the expression of the SCZ risk gene *BNIP3L* in the cerebellar hemisphere following multiple testing correction (P = 1.60E-05, adjusted P = 0.025). The *BNIP3L* gene is known to be involved in mitophagy, a process responsible for the selective removal of damaged mitochondria. Finally, to further explore genes with mitochondrial functions beyond the 120 prioritized SCZ genes, we performed a second trans eQTL analysis. This time we restricted the target genes to those within enriched GO terms related to mitochondrial function and energy production that had cell-type specific expression in the enriched cell-types from that earlier analysis. As a result, we tested the 10 LD-independent SNPs at MEF2C against 300 genes. Overall, the trans eQTL effect on the expression of *BNIP3L*, already detected, was the only finding that survived multiple test correction (S28 Table).

## Discussion

The present study aimed to integrate transcriptomic data from human neural cell models of *MEF2C* deletion with ChIP-seq data to identify the direct regulatory influence of *MEF2C* disruption on global transcriptional signatures. These data from models of early neuronal development stem cells (NSCs) and fully differentiated neurons (iNs) provide insight into the sets of genes downstream of *MEF2C* that may be important for brain function at different stages of neurodevelopment. Common variants in *MEF2C* are associated with SCZ and cognitive function. We do not expect these sets of downstream dysregulated genes to directly align with the molecular mechanisms of SCZ and cognitive function. But we have been able to interrogate these gene sets to determine if they were enriched for other genes associated with these phenotypes or other neurodevelopmental disorders. From there, we investigated the functionality of the genes within these sets to generate evidence that supports existing hypotheses about the molecular basis of SCZ.

All eight gene-sets were significantly enriched for genes associated with at least one phenotype but three gene-sets (NSCs_DELhom, iNs_DELhet_PB, and iNs_DELhom_PB) were enriched for common variants associated with SCZ, IQ and EA and were further enriched for rare DNMs reported in ID and/or DD patients. We also showed using PRS analysis that genetic risk for SCZ in each of these gene-sets could explain a significant proportion of variance in IQ. These data support a role for the genes in these sets in the aetiology of SCZ risk and associated cognitive dysfunction. Functional enrichment analysis has indicated that genes regulated by *MEF2C* may have a dual function in neurodevelopment. In the early stages, they are implicated in neuron generation, differentiation and development, and metabolic processes, while in later stages, these genes are involved in mitochondrial function and energy production.

The process of neurogenesis forms the fundamental basis of brain development, involving the differentiation of NSCs and neural progenitor cells (NPCs) into mature neurons [48]. NSCs have the capacity to differentiate into various functional neural lineage cells, such as neurons, astrocytes, and oligodendrocytes [49]. Aberrant neurogenesis from NSCs has been implicated as a potential underlying mechanism in the development of neuropsychiatric disorders [50,51]. This critical process appears to be susceptible to various genetic and environmental disruptions during early brain development. The cell-type enrichment analysis of our NSCs gene-sets indicated that their constituent genes are enriched within cycling progenitor cells and intermediate progenitor cells in the prenatal brain, which can produce new types of neurons and glial cells. GO analysis of the NSCs gene-sets also indicated a role for *MEF2C*-regulated genes in neuron development and differentiation. These findings suggest that the differentiation process from NSCs to these specific neuronal subtypes may be influenced by *MEF2C* disruption, and variants within the genes that encode this process may contribute to SCZ risk and cognitive dysfunction. Dysregulation in the normal development and functioning of these neural lineage cells and imbalance between them have been strongly linked to the underlying causes of SCZ and other neuropsychiatric disorders [52,53]. Enrichment analysis of KEGG pathways identified an enrichment of MEF2C direct target genes in NSCs within *Orexin receptor pathway*. The regulatory role of orexin (OXA) extends to various functions including sleep-wake rhythms, attention, cognition, and energy balance, all of which exhibit significant alterations in individuals with SCZ. Research has found inconsistent links between the OXA system and SCZ. Schizophrenia patients show decreased OXA plasma levels and hypothalamic OXA, with lower cortical OX2R mRNA in females. Conversely, males exhibit higher cortical OX1R and OX2R mRNA levels [54]. Furthermore, elevated OXA plasma levels have been associated with negative and disorganized symptoms in some studies [55]. We also observed that both glutamatergic excitatory neurons and GABAergic inhibitory interneurons in the prenatal and adult brain were enriched for genes from this NSCs set and from the two iNs sets. This provides further support for the balance of excitatory and inhibitory synapses, which is affected by *MEF2C* disruption [11], representing a potential molecular mechanism for neurodevelopmental disorders.

Synaptic activity is known to be an energy-intensive process that relies heavily on adenosine triphosphate (ATP) produced through oxidative phosphorylation (OXPHOS) in mitochondria [56]. OXPHOS involves the activity of electron transport chain (ETC) complexes (Complex I, II, III, and IV) and ATP synthase (Complex V), where electrons produced by the citric acid cycle are transferred across mitochondrial respiratory complexes [57]. Mitochondrial ATP production is crucial for various neuronal functions, including the assembly of the actin cytoskeleton for growth cone formation, development of pre-synaptic compartments, generation of membrane potential, and synaptic vesicle recycling and endocytosis. These processes contribute to essential synaptic activities and neuronal communication [58–60]. GO analysis of the iNs gene-sets identified that *MEF2C* directly regulates genes involved in ATP production, including those associated with OXPHOS.

Mitochondrial dysfunction has been implicated in the complex genetic mechanisms underlying SCZ. A total of 295 mitochondria-related genes associated with SCZ were identified through the examination of various studies encompassing copy number variants (CNVs), rare and *de novo* mutations, genome-wide associated SNPs, transcriptomic and proteomic studies of brain tissue from SCZ patients (reviewed in [61]). Significant associations were identified between SCZ and 19 nuclear mitochondria-related genes using GWAS data [62]. Four of these genes (*SMDT1*, *HSPE1*, *COQ10B*, and *FOXO3*) are in our iNs gene-sets. *FOXO3*, which is also significantly associated with IQ [8] is a transcription factor. It can translocate to the mitochondria where it may bind to mtDNA and react with mitochondrial transcription factor A (*TFAM*) and mitochondrial RNA polymerase (mtRNApol), inducing the production of various mitochondrial genes necessary for OXPHOS [63]. Large-scale brain eQTL studies have shown significant enrichment of mitochondria-related genes. Approximately 28% of the eQTL genes implicated in SCZ were related to mitochondria [64]. Furthermore, studies investigating gene expression in postmortem brain tissues of individuals with SCZ have consistently revealed a reduction in the expression of mitochondria-related genes. Specifically, genes such as NADH: ubiquinone oxidoreductase core subunit V1 (*NDUFV1*), NADH: ubiquinone oxidoreductase core subunit V2 (*NDUFV2*), NADH: ubiquinone oxidoreductase core subunit S1 (*NDUFS1*) [65–67], and cytochrome c oxidase (*COX*) show decreased expression levels in a region-specific manner [68]. *NDUFV2* and multiple isoforms of COX are present in the iNs gene-sets.

One of the most well established CNVs associated with SCZ is the deletion of chromosome 22q11.2, also known as 22q11.2 deletion syndrome (22q11.2DS) [69]. Individuals with 22q11DS often encounter cognitive impairments and a variety of neuropsychiatric disorders, including attention deficit hyperactivity disorder (ADHD), SCZ, anxiety, and ASD [69]. Among the genes deleted in 22q11DS, six (*MRPL40*, *PRODH*, *SLC25A1*, *TXNRD2*, *T10*, and *ZDHHC8*) encode for mitochondrial proteins, and 3 others (*COMT*, *UFD1L*, and *DGCR8*) have an indirect effect on mitochondrial function [70]. A recent study demonstrated mitochondrial deficits in iPSC-derived neurons from individuals with 22q11DS and SCZ. These deficits included reduced ATP levels, impaired activity of ETC complexes I and IV, and decreased levels of mitochondrial-translated proteins [71]. Our study revealed the direct regulatory influence of *MEF2C* on two mitochondrial-related genes (*TXNRD2* and *COMT*) located within the 22q11.2 region in iNs. *TXNRD2* encodes for the mitochondrial Thioredoxin Reductase 2, an enzyme that is essential for reactive oxygen species clearance in brain. In 22q model transgenic mice, mitochondrial TXNRD2 has been shown to impact synaptic function and is associated with long-range cortical connectivity and psychosis-related cognitive deficits [72]. A recent investigation demonstrated that an amyotrophic lateral sclerosis (ALS)-associated SNP located in the intronic region of MEF2C (rs304152), residing in a putative enhancer element, causes neuronal mitochondrial dysfunction. This dysfunction is characterized by decreased mitochondrial gene expression, impaired ATP production, increased oxidative stress, and decreased mitochondrial membrane potential [73]. Additionally, mitochondrial dysfunction can contribute to an imbalance in the excitatory (glutamate) and inhibitory (GABA) neurotransmitter systems [74–76], which we have referenced already as a potential molecular mechanism of neurodevelopmental disorder.

The most recent GWAS of SCZ prioritized 120 genes from the 287 genome-wide significant loci [7]. We identified a trans eQTL effect of a SNP in *MEF2C* on the expression of one of these prioritized genes, *BNIP3L*. Disruption of *MEF2C* in the iNs cell line resulted in reduced expression of *BNIP3L*. *BNIP3L* is involved in the selective removal of damaged mitochondria through a process called mitophagy. *BNIP3L* downregulation induces synaptic dysfunction arising from the accumulation of damaged mitochondria that leads to reduced mitochondrial respiration function and synaptic density [77]. It has been reported that mitophagy is significantly impaired in neurodegenerative disorders including Alzheimer’s disease, Parkinson’s disease, amyotrophic lateral sclerosis and Huntington’s [78–81]. A recent investigation has identified both common and rare mutations in the *BNIP3L* gene in individuals diagnosed with SCZ [82]. The effect of identified genome-wide significant SNPs at *MEF2C* on its function remains to be elucidated but here is evidence that these variants may have downstream effects on direct targets of *MEF2C*, in this case potentially dysregulating *BNIP3L* and potentially contributing to mitochondrial dysfunction.

A first limitation of this study is that the ChIP-seq data was not generated from the same human neural cell models as the RNA-seq data, it came from human fetal brain cultures. Ideally, these data would come from the same source when trying to combine them to identify direct target genes. In addition, it would have strengthened the study to have validated the ChIP-seq and RNA-seq results at some target genes with quantitative PCR. It is noteworthy that the three gene-sets that were enriched for common and rare variants associated with neurodevelopmental disorders and phenotypes were also the largest gene-sets (all >1,000 genes) whereas the other 5 gene-sets each contained <500 genes. Therefore, we likely had greater statistical power to detect enrichments in these larger gene-sets. The smaller gene-sets were all enriched for variants associated with SCZ, IQ or EA at least nominally significant levels and thus may also index relevant functions to these phenotypes.

In conclusion, our study leverages data from human neural cell models of *MEF2C* to investigate putative molecular mechanisms of SCZ and cognitive dysfunction. These include neuron development, metabolic processes and mitochondrial dysfunction including impaired ATP production, synaptic dysfunction, imbalance in neurotransmitter systems, and disrupted mitophagy. These mechanisms provide valuable insights into how *MEF2C* dysregulation could contribute to the development of these complex disorders. Further investigations into the precise molecular mechanisms by which *MEF2C* and mitochondrial genes contribute to the development of these disorders are needed. Such insights may pave the way for the development of novel therapeutic strategies targeting mitochondrial pathways in the treatment of neuropsychiatric disorders.

## Supporting information

S1 Fig Overlap of MEF2C direct target genes in NSCs (A) and iNs (B) for different mutations and genotypes. Venn diagrams illustrate the number of shared and unique MEF2C direct target genes in each cell type separately, across different genotypes and for different types of MEF2C gene mutation. NSCs: Neural stem cells; iNs: Induced neurons; DELhom: Homozygous deletion; DELhet: Heterozygous deletion; PB: Proximal boundary (indirect mutation of MEF2C).
(TIF)

S1 Table Significant DEGs in MEF2C-Disrupted NSCs and iNs. (XLSX)

S2 Table Direct Target Genes of MEF2C in NSCs and iNs Determined Using BETA Analysis. (XLSX)

S3 Table Genes Identified as Associated with SCZ (Trubetskoy et al. 2022) [7], IQ (Savage et al. 2018)[8] and EA (Lee et al. 2018) [9] by GWAS Analysis. (XLSX)

S4 Table Exome Sequencing Studies Reporting DMNs Used in this Study. (XLSX)

S5 Table Background Gene Lists Used for Gene Ontology and KEGG Pathway Enrichment Analysis. (XLSX)

S6 Table MEF2C ChIP-Seq Peak Annotation. (XLSX)

S7 Table Gene Ontology Analysis for Genes Proximal to MEF2C Peaks Identified via ChIP-seq Analysis. (XLSX)

S8 Table sLDSC Analysis Results of MEF2C Direct Target Gene-Sets Using GWAS Data for SCZ, EA, and IQ. (XLSX)

S9 Table sLDSC Analysis Results of MEF2C Direct Target Gene-Sets Using GWAS Data for SCZ, EA, and IQ, Excluding Genes Associated with IQ/EA or SCZ. (XLSX)

S10 Table sLDSC Analysis Results of MEF2C Direct Target Gene-Sets Using GWAS Data for Control Phenotypes. (XLSX)

S11 Table Gene-Set Based PRS Analysis of MEF2C Direct Target Gene-sets at Five Different PRS P-value Thresholds. (XLSX)

S12 Table Gene-Set Based PRS Analysis of MEF2C Direct Target Gene-sets at Five Different PRS P-value Thresholds, Excluding All Genes Associated with IQ or EA from the Gene-Set. (XLSX)

S13 Table Analysis of MEF2C Direct Target gene-sets Using Data on DNMs from Patients with SCZ, ASD, ID, and DD. (XLSX)

S14 Table EWCE analysis of MEF2C Direct Traget Gene-Sets in the Cameron et al. 2023 [42] scRNA-seq Data for 5 Brain Regions from Prenatal Brain. (XLSX)

S15 Table EWCE analysis of MEF2C Direct Target Gene-Sets Using Available Data From the Allen Brain Atlas (Human Multiple Cortical Areas SMART-seq) from Adult Human Brain. (XLSX)

S16 Table Gene Ontology and Pathway Enrichment Analysis for MEF2C Direct Target Gene-sets Generated from NSC_DELhet. (XLSX)

S17 Table Gene Ontology and Pathway Enrichment Analysis for MEF2C Direct Target Gene-sets Generated from NSC_DELhom. (XLSX)

S18 Table Gene Ontology and Pathway Enrichment Analysis for MEF2C Direct Target Gene-sets Generated from NSC_DELhet_PB. (XLSX)

S19 Table Gene Ontology and Pathway Enrichment Analysis for MEF2C Direct Target Gene-sets Generated from NSC_DELhom_PB. (XLSX)

S20 Table Gene Ontology and Pathway Enrichment Analysis for MEF2C Direct Target Gene-sets Generated from iNs_DELhet. (XLSX)

S21 Table Gene Ontology and Pathway Enrichment Analysis for MEF2C Direct Target Gene-sets Generated from iNs_DELhom. (XLSX)

S22 Table Gene Ontology and Pathway Enrichment Analysis for MEF2C Direct Target Gene-sets Generated from iNs_DELhet_PB. (XLSX)

S23 Table Gene Ontology and Pathway Enrichment Analysis for MEF2C Direct Target Gene-sets Generated from iNs_DELhom_PB. (XLSX)

S24 Table List of Associated Genes Found in the Enriched GO Terms. (XLSX)

S25 Table Genes Prioritized in the Latest GWAS for SCZ (Trubetskoy et al. 2022) [7]. (XLSX)

S26 Table List of Genes Overlapping between GO Term Enriched Genes (S24 Table) and Genes Prioritized in the Latest SCZ GWAS (S25 Table). (XLSX)

S27 Table List of Independent Genome-Wide Significant SNPs at MEF2C SNPs Associated with SCZ, EA, and IQ. (XLSX)

S28 Table eQTL Analysis for the LD-Independent MEF2C SNPs on MEF2C Direct Target Genes Associated with Mitochondrial Function and Cell-Type Specific Expression in Different Brain Tissues Based on the GTEx Dataset. (XLSX)

## References

[1] Leifer D, Golden J, Kowall NW: Myocyte-specific enhancer binding factor 2C expression in human brain development. *Neuroscience* 1994, 63:1067–1079. doi: 10.1016/0306-4522(94)90573-8 7700509

[2] Lyons GE, Micales BK, Schwarz J, Martin JF, Olson EN: Expression of mef2 genes in the mouse central nervous system suggests a role in neuronal maturation. *J Neurosci* 1995, 15:5727–5738. doi: 10.1523/JNEUROSCI.15-08-05727.1995 7643214 PMC6577647

[3] Li H, Radford JC, Ragusa MJ, Shea KL, McKercher SR, Zaremba JD, Soussou W, Nie Z, Kang Y-J, Nakanishi N, et al.: Transcription factor MEF2C influences neural stem/progenitor cell differentiation and maturation in vivo. *Proceedings of the National Academy of Sciences* 2008, 105:9397–9402. doi: 10.1073/pnas.0802876105 18599437 PMC2453715

[4] UniProt Consortium: UniProt: a hub for protein information. *Nucleic Acids Res* 2015, 43:D204–212. 25348405 10.1093/nar/gku989 PMC4384041

[5] Potthoff MJ, Olson EN: MEF2: a central regulator of diverse developmental programs. *Development* 2007, 134:4131–4140. doi: 10.1242/dev.008367 17959722

[6] Engels H, Wohlleber E, Zink A, Hoyer J, Ludwig KU, Brockschmidt FF, Wieczorek D, Moog U, Hellmann-Mersch B, Weber RG, et al.: A novel microdeletion syndrome involving 5q14.3-q15: clinical and molecular cytogenetic characterization of three patients. *Eur J Hum Genet* 2009, 17:1592–1599. doi: 10.1038/ejhg.2009.90 19471318 PMC2987012

[7] Trubetskoy V, Pardiñas AF, Qi T, Panagiotaropoulou G, Awasthi S, Bigdeli TB, Bryois J, Chen C-Y, Dennison CA, Hall LS, et al.: Mapping genomic loci implicates genes and synaptic biology in schizophrenia. *Nature* 2022, 604:502–508. doi: 10.1038/s41586-022-04434-5 35396580 PMC9392466

[8] Savage JE, Jansen PR, Stringer S, Watanabe K, Bryois J, de Leeuw CA, Nagel M, Awasthi S, Barr PB, Coleman JRI, et al.: Genome-wide association meta-analysis in 269,867 individuals identifies new genetic and functional links to intelligence. *Nat Genet* 2018, 50:912–919. doi: 10.1038/s41588-018-0152-6 29942086 PMC6411041

[9] Lee JJ, Wedow R, Okbay A, Kong E, Maghzian O, Zacher M, Nguyen-Viet TA, Bowers P, Sidorenko J, Karlsson Linnér R, et al.: Gene discovery and polygenic prediction from a genome-wide association study of educational attainment in 1.1 million individuals. *Nat Genet* 2018, 50:1112–1121. doi: 10.1038/s41588-018-0147-3 30038396 PMC6393768

[10] Mitchell AC, Javidfar B, Pothula V, Ibi D, Shen EY, Peter CJ, Bicks LK, Fehr T, Jiang Y, Brennand KJ, et al.: MEF2C transcription factor is associated with the genetic and epigenetic risk architecture of schizophrenia and improves cognition in mice. *Mol Psychiatry* 2018, 23:123–132. doi: 10.1038/mp.2016.254 28115742 PMC5966823

[11] Harrington AJ, Raissi A, Rajkovich K, Berto S, Kumar J, Molinaro G, Raduazzo J, Guo Y, Loerwald K, Konopka G, et al.: MEF2C regulates cortical inhibitory and excitatory synapses and behaviors relevant to neurodevelopmental disorders. *eLife* 2016, 5:e20059. doi: 10.7554/eLife.20059 27779093 PMC5094851

[12] Harrington AJ, Bridges CM, Berto S, Blankenship K, Cho JY, Assali A, Siemsen BM, Moore HW, Tsvetkov E, Thielking A, et al.: MEF2C Hypofunction in Neuronal and Neuroimmune Populations Produces MEF2C Haploinsufficiency Syndrome-like Behaviors in Mice. *Biol Psychiatry* 2020, 88:488–499. doi: 10.1016/j.biopsych.2020.03.011 32418612 PMC7483399

[13] Adachi M, Lin P-Y, Pranav H, Monteggia LM: Postnatal Loss of Mef2c Results in Dissociation of Effects on Synapse Number and Learning and Memory. *Biol Psychiatry* 2016, 80:140–148. doi: 10.1016/j.biopsych.2015.09.018 26642739 PMC4826326

[14] Tu S, Akhtar MW, Escorihuela RM, Amador-Arjona A, Swarup V, Parker J, Zaremba JD, Holland T, Bansal N, Holohan DR, et al.: NitroSynapsin therapy for a mouse MEF2C haploinsufficiency model of human autism. *Nat Commun* 2017, 8:1488. doi: 10.1038/s41467-017-01563-8 29133852 PMC5684358

[15] Barbosa AC, Kim M-S, Ertunc M, Adachi M, Nelson ED, McAnally J, Richardson JA, Kavalali ET, Monteggia LM, Bassel-Duby R, et al.: MEF2C, a transcription factor that facilitates learning and memory by negative regulation of synapse numbers and function. *Proceedings of the National Academy of Sciences* 2008, 105:9391–9396. doi: 10.1073/pnas.0802679105 18599438 PMC2453723

[16] Mohajeri K, Yadav R, D’haene E, Boone PM, Erdin S, Gao D, Moyses-Oliveira M, Bhavsar R, Currall BB, O’Keefe K, et al.: Transcriptional and functional consequences of alterations to MEF2C and its topological organization in neuronal models. *Am J Hum Genet* 2022, 109:2049–2067. doi: 10.1016/j.ajhg.2022.09.015 36283406 PMC9674968

[17] Gasiorowska A, Wydrych M, Drapich P, Zadrozny M, Steczkowska M, Niewiadomski W, Niewiadomska G: The Biology and Pathobiology of Glutamatergic, Cholinergic, and Dopaminergic Signaling in the Aging Brain. *Front Aging Neurosci* 2021, 13:654931. doi: 10.3389/fnagi.2021.654931 34326765 PMC8315271

[18] Ataman B, Boulting GL, Harmin DA, Yang MG, Baker-Salisbury M, Yap E-L, Malik AN, Mei K, Rubin AA, Spiegel I, et al.: Evolution of Osteocrin as an activity-regulated factor in the primate brain. *Nature* 2016, 539:242–247. doi: 10.1038/nature20111 27830782 PMC5499253

[19] Li H, Durbin R: Fast and accurate short read alignment with Burrows–Wheeler transform. *Bioinformatics* 2009, 25:1754–1760. doi: 10.1093/bioinformatics/btp324 19451168 PMC2705234

[20] Li H, Handsaker B, Wysoker A, Fennell T, Ruan J, Homer N, Marth G, Abecasis G, Durbin R, 1000 Genome Project Data Processing Subgroup: The Sequence Alignment/Map format and SAMtools. *Bioinformatics* 2009, 25:2078–2079. 19505943 10.1093/bioinformatics/btp352 PMC2723002

[21] Zhang Y, Liu T, Meyer CA, Eeckhoute J, Johnson DS, Bernstein BE, Nusbaum C, Myers RM, Brown M, Li W, et al.: Model-based Analysis of ChIP-Seq (MACS). *Genome Biology* 2008, 9:R137. doi: 10.1186/gb-2008-9-9-r137 18798982 PMC2592715

[22] Yu G, Wang L-G, He Q-Y: ChIPseeker: an R/Bioconductor package for ChIP peak annotation, comparison and visualization. *Bioinformatics* 2015, 31:2382–2383. doi: 10.1093/bioinformatics/btv145 25765347

[23] Wang S, Sun H, Ma J, Zang C, Wang C, Wang J, Tang Q, Meyer CA, Zhang Y, Liu XS: Target analysis by integration of transcriptome and ChIP-seq data with BETA. *Nat Protoc* 2013, 8:2502–2515. doi: 10.1038/nprot.2013.150 24263090 PMC4135175

[24] Bulik-Sullivan BK, Loh P-R, Finucane HK, Ripke S, Yang J, Schizophrenia Working Group of the Psychiatric Genomics Consortium, Patterson N, Daly MJ, Price AL, Neale BM: LD Score regression distinguishes confounding from polygenicity in genome-wide association studies. *Nat Genet* 2015, 47:291–295. 25642630 10.1038/ng.3211 PMC4495769

[25] Demontis D, Walters RK, Martin J, Mattheisen M, Als TD, Agerbo E, Baldursson G, Belliveau R, Bybjerg-Grauholm J, Bækvad-Hansen M, et al.: Discovery of the first genome-wide significant risk loci for attention-deficit/hyperactivity disorder. *Nat Genet* 2019, 51:63–75. doi: 10.1038/s41588-018-0269-7 30478444 PMC6481311

[26] International Obsessive Compulsive Disorder Foundation Genetics Collaborative (IOCDF-GC) and OCD Collaborative Genetics Association Studies (OCGAS): Revealing the complex genetic architecture of obsessive-compulsive disorder using meta-analysis. *Mol Psychiatry* 2018, 23:1181–1188. 28761083 10.1038/mp.2017.154 PMC6660151

[27] Lambert JC, Ibrahim-Verbaas CA, Harold D, Naj AC, Sims R, Bellenguez C, DeStafano AL, Bis JC, Beecham GW, Grenier-Boley B, et al.: Meta-analysis of 74,046 individuals identifies 11 new susceptibility loci for Alzheimer’s disease. *Nat Genet* 2013, 45:1452–1458. doi: 10.1038/ng.2802 24162737 PMC3896259

[28] Traylor M, Farrall M, Holliday EG, Sudlow C, Hopewell JC, Cheng Y-C, Fornage M, Ikram MA, Malik R, Bevan S, et al.: Genetic risk factors for ischaemic stroke and its subtypes (the METASTROKE collaboration): a meta-analysis of genome-wide association studies. *Lancet Neurol* 2012, 11:951–962. doi: 10.1016/S1474-4422(12)70234-X 23041239 PMC3490334

[29] Corley E, Patlola SR, Laighneach A, Corvin A, McManus R, Kenyon M, Kelly JP, Mckernan DP, King S, Hallahan B, et al.: Genetic and inflammatory effects on childhood trauma and cognitive functioning in patients with schizophrenia and healthy participants. *Brain*, *Behavior*, *and Immunity* 2024, 115:26–37. doi: 10.1016/j.bbi.2023.09.013 37748567

[30] Whitton L, Cosgrove D, Clarkson C, Harold D, Kendall K, Richards A, Mantripragada K, Owen MJ, O’Donovan MC, Walters J, et al.: Cognitive analysis of schizophrenia risk genes that function as epigenetic regulators of gene expression. *American Journal of Medical Genetics Part B*: *Neuropsychiatric Genetics* 2016, 171:1170–1179. doi: 10.1002/ajmg.b.32503 27762073

[31] Ware JS, Samocha KE, Homsy J, Daly MJ: Interpreting de novo Variation in Human Disease Using denovolyzeR. *Curr Protoc Hum Genet* 2015, 87:7.25.1–7.25.15. doi: 10.1002/0471142905.hg0725s87 26439716 PMC4606471

[32] Howrigan DP, Rose SA, Samocha KE, Fromer M, Cerrato F, Chen WJ, Churchhouse C, Chambert K, Chandler SD, Daly MJ, et al.: Exome sequencing in schizophrenia-affected parent–offspring trios reveals risk conferred by protein-coding de novo mutations. *Nat Neurosci* 2020, 23:185–193. doi: 10.1038/s41593-019-0564-3 31932770 PMC7007385

[33] Wang Q, Li M, Yang Z, Hu X, Wu H-M, Ni P, Ren H, Deng W, Li M, Ma X, et al.: Increased co-expression of genes harboring the damaging de novo mutations in Chinese schizophrenic patients during prenatal development. *Sci Rep* 2015, 5:18209. doi: 10.1038/srep18209 26666178 PMC4678883

[34] Ambalavanan A, Girard SL, Ahn K, Zhou S, Dionne-Laporte A, Spiegelman D, Bourassa CV, Gauthier J, Hamdan FF, Xiong L, et al.: De novo variants in sporadic cases of childhood onset schizophrenia. *Eur J Hum Genet* 2016, 24:944–948. doi: 10.1038/ejhg.2015.218 26508570 PMC4867457

[35] Rees E, Han J, Morgan J, Carrera N, Escott-Price V, Pocklington AJ, Duffield M, Hall LS, Legge SE, Pardiñas AF, et al.: De novo mutations identified by exome sequencing implicate rare missense variants in SLC6A1 in schizophrenia. *Nat Neurosci* 2020, 23:179–184. doi: 10.1038/s41593-019-0565-2 31932766 PMC7007300

[36] Satterstrom FK, Kosmicki JA, Wang J, Breen MS, De Rubeis S, An J-Y, Peng M, Collins R, Grove J, Klei L, et al.: Large-Scale Exome Sequencing Study Implicates Both Developmental and Functional Changes in the Neurobiology of Autism. *Cell* 2020, 180:568–584.e23. doi: 10.1016/j.cell.2019.12.036 31981491 PMC7250485

[37] Genovese G, Fromer M, Stahl EA, Ruderfer DM, Chambert K, Landén M, Moran JL, Purcell SM, Sklar P, Sullivan PF, et al.: Increased burden of ultra-rare protein-altering variants among 4,877 individuals with schizophrenia. *Nat Neurosci* 2016, 19:1433–1441. doi: 10.1038/nn.4402 27694994 PMC5104192

[38] Bowling KM, Thompson ML, Amaral MD, Finnila CR, Hiatt SM, Engel KL, Cochran JN, Brothers KB, East KM, Gray DE, et al.: Genomic diagnosis for children with intellectual disability and/or developmental delay. *Genome Med* 2017, 9:43. doi: 10.1186/s13073-017-0433-1 28554332 PMC5448144

[39] Chevarin M, Duffourd Y, Barnard RA, Moutton S, Lecoquierre F, Daoud F, Kuentz P, Cabret C, Thevenon J, Gautier E, et al.: Excess of de novo variants in genes involved in chromatin remodelling in patients with marfanoid habitus and intellectual disability. *Journal of Medical Genetics* 2020, 57:466–474. doi: 10.1136/jmedgenet-2019-106425 32277047

[40] Deciphering Developmental Disorders Study: Prevalence and architecture of de novo mutations in developmental disorders. *Nature* 2017, 542:433–438. 28135719 10.1038/nature21062 PMC6016744

[41] Skene NG, Grant SGN: Identification of Vulnerable Cell Types in Major Brain Disorders Using Single Cell Transcriptomes and Expression Weighted Cell Type Enrichment. *Front Neurosci* 2016, 10:16. doi: 10.3389/fnins.2016.00016 26858593 PMC4730103

[42] Cameron D, Mi D, Vinh N-N, Webber C, Li M, Marín O, O’Donovan MC, Bray NJ: Single-Nuclei RNA Sequencing of 5 Regions of the Human Prenatal Brain Implicates Developing Neuron Populations in Genetic Risk for Schizophrenia. *Biological Psychiatry* 2023, 93:157. doi: 10.1016/j.biopsych.2022.06.033 36150908 PMC10804933

[43] Hodge RD, Bakken TE, Miller JA, Smith KA, Barkan ER, Graybuck LT, Close JL, Long B, Johansen N, Penn O, et al.: Conserved cell types with divergent features in human versus mouse cortex. *Nature* 2019, 573:61–68. doi: 10.1038/s41586-019-1506-7 31435019 PMC6919571

[44] Tasic B, Yao Z, Graybuck LT, Smith KA, Nguyen TN, Bertagnolli D, Goldy J, Garren E, Economo MN, Viswanathan S, et al.: Shared and distinct transcriptomic cell types across neocortical areas. *Nature* 2018, 563:72–78. doi: 10.1038/s41586-018-0654-5 30382198 PMC6456269

[45] Bindea G, Galon J, Mlecnik B: CluePedia Cytoscape plugin: pathway insights using integrated experimental and in silico data. *Bioinformatics* 2013, 29:661–663. doi: 10.1093/bioinformatics/btt019 23325622 PMC3582273

[46] Sjöstedt E, Zhong W, Fagerberg L, Karlsson M, Mitsios N, Adori C, Oksvold P, Edfors F, Limiszewska A, Hikmet F, et al.: An atlas of the protein-coding genes in the human, pig, and mouse brain. *Science* 2020, 367:eaay5947. doi: 10.1126/science.aay5947 32139519

[47] Lonsdale J, Thomas J, Salvatore M, Phillips R, Lo E, Shad S, Hasz R, Walters G, Garcia F, Young N, et al.: The Genotype-Tissue Expression (GTEx) project. *Nat Genet* 2013, 45:580–585. doi: 10.1038/ng.2653 23715323 PMC4010069

[48] Ribeiro FF, Xapelli S: An Overview of Adult Neurogenesis. In *Recent Advances in NGF and Related Molecules*: *The Continuum of the NGF “Saga*.*”* Edited by Calzà L, Aloe L, Giardino L. Springer International Publishing; 2021:77–94. 10.1007/978-3-030-74046-7_7 34453294

[49] Thier M, Wörsdörfer P, Lakes YB, Gorris R, Herms S, Opitz T, Seiferling D, Quandel T, Hoffmann P, Nöthen MM, et al.: Direct Conversion of Fibroblasts into Stably Expandable Neural Stem Cells. *Cell Stem Cell* 2012, 10:473–479. doi: 10.1016/j.stem.2012.03.003 22445518

[50] Hagihara H, Takao K, Walton NM, Matsumoto M, Miyakawa T: Immature Dentate Gyrus: An Endophenotype of Neuropsychiatric Disorders. Neural Plast 2013, 2013:318596. doi: 10.1155/2013/318596 23840971 PMC3694492

[51] Cho K-O, Lybrand ZR, Ito N, Brulet R, Tafacory F, Zhang L, Good L, Ure K, Kernie SG, Birnbaum SG, et al.: Aberrant hippocampal neurogenesis contributes to epilepsy and associated cognitive decline. *Nature Communications* 2015, 6:6606. doi: 10.1038/ncomms7606 25808087 PMC4375780

[52] Gao R, Penzes P: Common Mechanisms of Excitatory and Inhibitory Imbalance in Schizophrenia and Autism Spectrum Disorders. *Curr Mol Med* 2015, 15:146–167. doi: 10.2174/1566524015666150303003028 25732149 PMC4721588

[53] Foss-Feig JH, Adkinson BD, Ji JL, Yang G, Srihari VH, McPartland JC, Krystal JH, Murray JD, Anticevic A: Searching for Cross-Diagnostic Convergence: Neural Mechanisms Governing Excitation and Inhibition Balance in Schizophrenia and Autism Spectrum Disorders. *Biological Psychiatry* 2017, 81:848–861. doi: 10.1016/j.biopsych.2017.03.005 28434615 PMC5436134

[54] Lu J, Huang M-L, Li J-H, Jin K-Y, Li H-M, Mou T-T, Fronczek R, Duan J-F, Xu W-J, Swaab D, et al.: Changes of Hypocretin (Orexin) System in Schizophrenia: From Plasma to Brain. *Schizophr Bull* 2021, 47:1310–1319. doi: 10.1093/schbul/sbab042 33974073 PMC8379539

[55] Chien Y-L, Liu C-M, Shan J-C, Lee H-J, Hsieh MH, Hwu H-G, Chiou L-C: Elevated plasma orexin A levels in a subgroup of patients with schizophrenia associated with fewer negative and disorganized symptoms. *Psychoneuroendocrinology* 2015, 53:1–9. doi: 10.1016/j.psyneuen.2014.12.012 25560205

[56] Hall CN, Klein-Flügge MC, Howarth C, Attwell D: Oxidative Phosphorylation, Not Glycolysis, Powers Presynaptic and Postsynaptic Mechanisms Underlying Brain Information Processing. *J Neurosci* 2012, 32:8940–8951. doi: 10.1523/JNEUROSCI.0026-12.2012 22745494 PMC3390246

[57] Wen Y: Maxwell’s demon at work: Mitochondria, the organelles that convert information into energy? *Chronic Dis Transl Med* 2018, 4:135–138. 29988942 10.1016/j.cdtm.2018.05.002 PMC6034008

[58] Verstreken P, Ly CV, Venken KJT, Koh T-W, Zhou Y, Bellen HJ: Synaptic Mitochondria Are Critical for Mobilization of Reserve Pool Vesicles at Drosophila Neuromuscular Junctions. *Neuron* 2005, 47:365–378. doi: 10.1016/j.neuron.2005.06.018 16055061

[59] Attwell D, Laughlin SB: An Energy Budget for Signaling in the Grey Matter of the Brain. *Journal of Cerebral Blood Flow & Metabolism* 2001, 21:1133. doi: 10.1097/00004647-200110000-00001 11598490

[60] Lee CW, Peng HB: The function of mitochondria in presynaptic development at the neuromuscular junction. *Mol Biol Cell* 2008, 19:150–158. doi: 10.1091/mbc.e07-05-0515 17942598 PMC2174173

[61] Hjelm BE, Rollins B, Mamdani F, Lauterborn JC, Kirov G, Lynch G, Gall CM, Sequeira A, Vawter MP: Evidence of Mitochondrial Dysfunction within the Complex Genetic Etiology of Schizophrenia. *Mol Neuropsychiatry* 2015, 1:201–219. doi: 10.1159/000441252 26550561 PMC4635522

[62] Gonçalves VF, Cappi C, Hagen CM, Sequeira A, Vawter MP, Derkach A, Zai CC, Hedley PL, Bybjerg-Grauholm J, Pouget JG, et al.: A comprehensive analysis of nuclear-encoded mitochondrial genes in schizophrenia. *Biol Psychiatry* 2018, 83:780–789. doi: 10.1016/j.biopsych.2018.02.1175 29628042 PMC7168759

[63] Celestini V, Tezil T, Russo L, Fasano C, Sanese P, Forte G, Peserico A, Lepore Signorile M, Longo G, De Rasmo D, et al.: Uncoupling FoxO3A mitochondrial and nuclear functions in cancer cells undergoing metabolic stress and chemotherapy. *Cell Death Dis* 2018, 9:231. doi: 10.1038/s41419-018-0336-0 29445193 PMC5833443

[64] Kim Y, Xia K, Tao R, Giusti-Rodriguez P, Vladimirov V, van den Oord E, Sullivan PF: A meta-analysis of gene expression quantitative trait loci in brain. *Transl Psychiatry* 2014, 4:e459. doi: 10.1038/tp.2014.96 25290266 PMC4350525

[65] Ben-Shachar D, Karry R: Neuroanatomical Pattern of Mitochondrial Complex I Pathology Varies between Schizophrenia, Bipolar Disorder and Major Depression. *PLOS ONE* 2008, 3:e3676. doi: 10.1371/journal.pone.0003676 18989376 PMC2579333

[66] Ben-Shachar D, Karry R: Sp1 Expression Is Disrupted in Schizophrenia; A Possible Mechanism for the Abnormal Expression of Mitochondrial Complex I Genes, NDUFV1 and NDUFV2. *PLOS ONE* 2007, 2: e817. doi: 10.1371/journal.pone.0000817 17786189 PMC1950689

[67] Ben-Shachar D: Mitochondrial complex I as a possible novel peripheral biomarker for schizophrenia. In *The handbook of neuropsychiatric biomarkers*, *endophenotypes and genes*, *Vol 3*: *Metabolic and peripheral biomarkers*. Springer Science + Business Media; 2009:71–83.

[68] Rice MW, Smith KL, Roberts RC, Perez-Costas E, Melendez-Ferro M: Assessment of Cytochrome C Oxidase Dysfunction in the Substantia Nigra/Ventral Tegmental Area in Schizophrenia. *PLOS ONE* 2014, 9:e100054. doi: 10.1371/journal.pone.0100054 24941246 PMC4062438

[69] McDonald-McGinn DM, Sullivan KE, Marino B, Philip N, Swillen A, Vorstman JAS, Zackai EH, Emanuel BS, Vermeesch JR, Morrow BE, et al.: 22q11.2 deletion syndrome. *Nat Rev Dis Primers* 2015, 1:15071. doi: 10.1038/nrdp.2015.71 27189754 PMC4900471

[70] Napoli E, Tassone F, Wong S, Angkustsiri K, Simon TJ, Song G, Giulivi C: Mitochondrial Citrate Transporter-dependent Metabolic Signature in the 22q11.2 Deletion Syndrome. *J Biol Chem* 2015, 290:23240–23253. doi: 10.1074/jbc.M115.672360 26221035 PMC4645608

[71] Li J, Ryan SK, Deboer E, Cook K, Fitzgerald S, Lachman HM, Wallace DC, Goldberg EM, Anderson SA: Mitochondrial deficits in human iPSC-derived neurons from patients with 22q11.2 deletion syndrome and schizophrenia. *Transl Psychiatry* 2019, 9:302. 10.1038/s41398-019-0643-y PMC6861238 31740674

[72] Fernandez A, Meechan DW, Karpinski BA, Paronett EM, Bryan CA, Rutz HL, Radin EA, Lubin N, Bonner ER, Popratiloff A, et al.: Mitochondrial Dysfunction Leads to Cortical Under-connectivity and Cognitive Impairment. *Neuron* 2019, 102:1127–1142.e3. doi: 10.1016/j.neuron.2019.04.013 31079872 PMC6668992

[73] Yousefian-Jazi A, Kim S, Choi S-H, Chu J, Nguyen PT-T, Park U, Lim K, Hwang H, Lee K, Kim Y, et al.: Loss of MEF2C function by enhancer mutation leads to neuronal mitochondria dysfunction and motor deficits in mice. 2024, doi: 10.1101/2024.07.15.603186 39071309 PMC11275751

[74] Anderson M, Hooker BS, Herbert M: Bridging from Cells to Cognition in Autism Pathophysiology: Biological Pathways to Defective Brain Function and Plasticity. *American Journal of Biochemistry and Biotechnology*, 4(2):167–176 2008, 4.

[75] Rubenstein JLR, Merzenich MM: Model of autism: increased ratio of excitation/inhibition in key neural systems. *Genes Brain Behav* 2003, 2:255–267. doi: 10.1034/j.1601-183x.2003.00037.x 14606691 PMC6748642

[76] Liu Y, Ouyang P, Zheng Y, Mi L, Zhao J, Ning Y, Guo W: A Selective Review of the Excitatory-Inhibitory Imbalance in Schizophrenia: Underlying Biology, Genetics, Microcircuits, and Symptoms. *Front Cell Dev Biol* 2021, 9:664535. 10.3389/fcell.2021.664535 PMC8567014 34746116

[77] Choi GE, Lee HJ, Chae CW, Cho JH, Jung YH, Kim JS, Kim SY, Lim JR, Han HJ: BNIP3L/NIX-mediated mitophagy protects against glucocorticoid-induced synapse defects. *Nat Commun* 2021, 12:487. doi: 10.1038/s41467-020-20679-y 33473105 PMC7817668

[78] Fang EF, Hou Y, Palikaras K, Adriaanse BA, Kerr JS, Yang B, Lautrup S, Hasan-Olive MM, Caponio D, Dan X, et al.: Mitophagy inhibits amyloid-β and tau pathology and reverses cognitive deficits in models of Alzheimer’s disease. *Nat Neurosci* 2019, 22:401–412. 30742114 10.1038/s41593-018-0332-9 PMC6693625

[79] Chu CT: Multiple Pathways for Mitophagy: A Neurodegenerative Conundrum for Parkinson’s Disease. *Neurosci Lett* 2019, 697:66–71. doi: 10.1016/j.neulet.2018.04.004 29626647 PMC6170746

[80] Palomo GM, Granatiero V, Kawamata H, Konrad C, Kim M, Arreguin AJ, Zhao D, Milner TA, Manfredi G: Parkin is a disease modifier in the mutant SOD1 mouse model of ALS. *EMBO Mol Med* 2018, 10:e8888. doi: 10.15252/emmm.201808888 30126943 PMC6180298

[81] Hwang S, Disatnik M-H, Mochly-Rosen D: Impaired GAPDH-induced mitophagy contributes to the pathology of Huntington’s disease. *EMBO Mol Med* 2015, 7:1307–1326. doi: 10.15252/emmm.201505256 26268247 PMC4604685

[82] Zhou J, Ma C, Wang K, Li X, Jian X, Zhang H, Yuan J, Yin J, Chen J, Shi Y: Identification of rare and common variants in BNIP3L: a schizophrenia susceptibility gene. *Hum Genomics* 2020, 14:16. doi: 10.1186/s40246-020-00266-4 32393399 PMC7212671

