# Supplementary figures and images for: Direct targets of MEF2C are enriched for genes associated with schizophrenia and cognitive function and are involved in neuron development and mitochondrial function
Source: PLoS Genet. 2024 Sep 11;20(9):e1011093. doi: 10.1371/journal.pgen.1011093 (PMC11419381; doi:10.1371/journal.pgen.1011093)

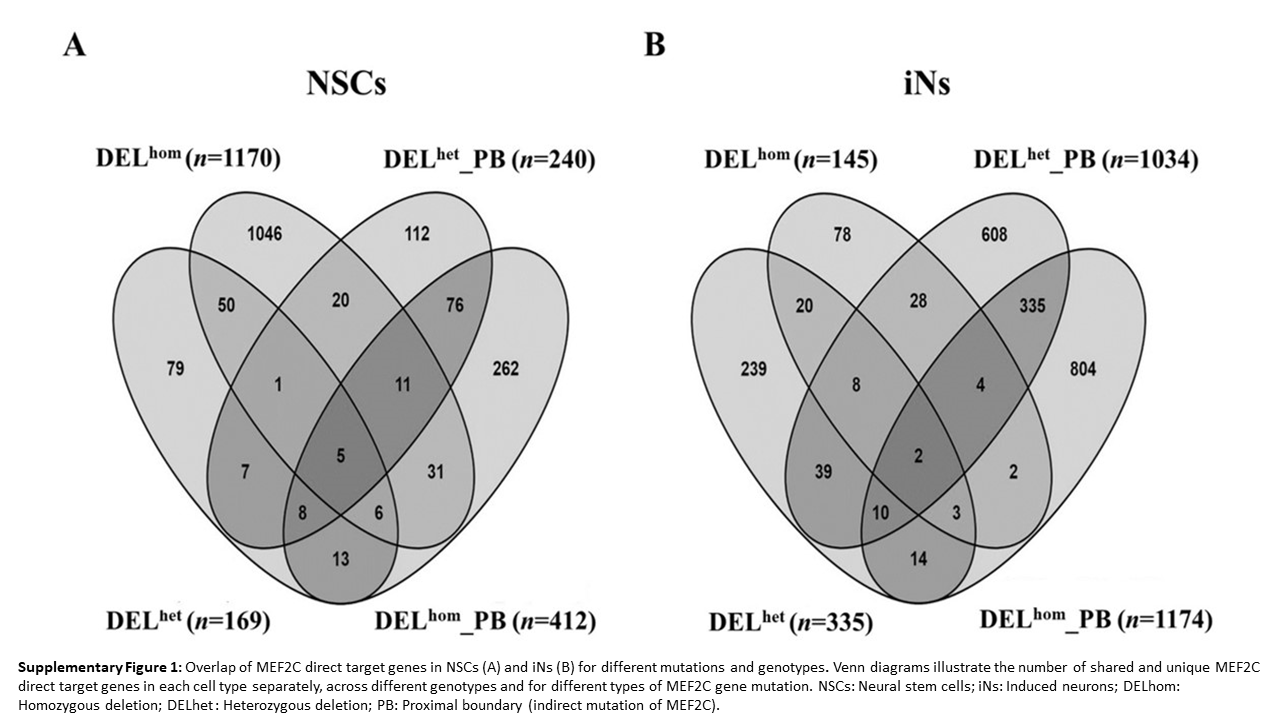

Supplement: S1 Fig — Venn diagrams illustrate the number of shared and unique MEF2C direct target genes in each cell type separately, across different genotypes and for different types of MEF2C gene mutation. NSCs: Neural stem cells; iNs: Induced neurons; DELhom: Homozygous deletion; DELhet: Heterozygous deletion; PB: Proximal boundary (indirect mutation of MEF2C). (TIF) [file pgen.1011093.s001.tif]
