# Supplementary material for: Direct targets of MEF2C are enriched for genes associated with schizophrenia and cognitive function and are involved in neuron development and mitochondrial function
Source: PLoS Genet. 2024 Sep 11;20(9):e1011093. doi: 10.1371/journal.pgen.1011093 (PMC11419381; doi:10.1371/journal.pgen.1011093)
Supplement: Attachment — Submitted filename: Responses to Reviewers Comments.docx [file pgen.1011093.s030.docx]

**Responses to Reviewers’ Comments:**

**Reviewer #1:**

This is a well-written manuscript exploring enrichment of genetic variation associated with schizophrenia and cognitive function in genomic targets of MEF2C in human neural stem cells and induced glutamatergic neurons. Variation in the MEF2C gene is itself associated with schizophrenia, cognition and developmental delay, and the finding that targets of MEF2C are also enriched for associations with these traits - and elucidation of the cells and mechanisms through which they operate - is very interesting. The authors use data from a recent study exploring transcriptional consequences of MEF2C perturbation (either direct deletion of deletion of the proximal boundary) in these cells (Mohajeri et al, AJHG 2022) and, importantly, intersect these with MEF2C ChIP-Seq data from human foetal brain to base their analyses on likely direct MEF2C targets. Their identification of a putative trans-eQTL effect of MEF2C genetic variation on prioritized schizophrenia risk gene BNIP3L is also of interest. The study appears to be carried out carefully, with a Discussion that nicely places the work in the context of existing literature.

I have the following suggestions for improvement of the manuscript:

**(1.1)** The manuscript appears to be missing a dedicated Methods section. Although the methods are described to some extent in the Results (and Figure 1 provides a nice overview of the methodology employed), I think more detail of the following is necessary:

- The GWAS data used for the analyses. I’m guessing these are from refs 7-9, but these are not referenced in the relevant Results section (page 14). A dedicated methods section could also state where these data were downloaded from.

- The ChIP-Seq data.

- The SLDSR analyses (e.g. did the authors use the baseline model to derive their P-values? Are P-values 1- or 2-tailed? [the former OK as only testing enrichment].)

- The exome sequencing data for ASD, ID and DD. This should be referenced in the relevant Results section (page 18) and the Methods section could indicate where these data were downloaded from.

- Gene Ontology analyses – which tool was used for this?

- Cell type enrichment analysis - how was this carried out?

***Response 1.1***

*We took the unconventional approach for Plos Genetics and included our dedicated Methods section after the Introduction and before the Results, as opposed to placing it at the end of the paper. Therefore, there was a full Methods section included in the original submission. To address the specific comments above, we have also now:*

*- Referenced the source of GWAS data and de novo mutation data in the Results (using the same references as supplied in the Methods section). The Methods also clearly states where these data have been downloaded from and a new S4 Table has been added with this information.*

*- Described the ChIP-Seq data in the Methods and more information about these data has been supplied in both the Methods and the Results in response to other comments made by Reviewer 2 below.*

*- Confirmed for the LDSC analysis that yes, the baseline model was used to derive P-values. The original Methods stated that “Enrichment of heritability was assessed controlling for the effects of 53 functional annotations included in the full baseline model version. Enrichment for heritability was compared to the baseline model using a corresponding P value.” And we have added that P values were “**one-tailed to identify significant local SNP-heritability”.*

*- Confirmed that the GO analyses are described in the original Methods but we have now also mentioned the method (with references) in the Results.*

*- Confirmed that the cell type enrichment analyses are described in the original Methods but we have now also mentioned the method (with references) in the Results.*

**(1.2)** Page 5: ‘iNs are responsible for synthesizing glutamate….’ Suggest ‘Glutamatergic iNs synthesize glutamate…’ instead (as iNs can also be GABAergic etc).

***Response 1.2***

*The text has been edited as suggested.*

**(1.3)** Figure 1: typo ‘human feta brain tissue’

***Response 1.3***

*The text has been corrected.*

**(1.4)** Page 18: ‘ASD patients’. Suggest ‘people with autism’ instead.

***Response 1.4***

*The text has been edited as suggested.*

**(1.5)** Page 25: Adult hippocampal neurogenesis in humans is still controversial, so this could be acknowledged.

***Response 1.5***

*The sentence has now been deleted.*

**(1.6)** Page 29. The authors acknowledge that use of single cell data from the adult mouse brain precluded analyses in cells from an early stage of neurodevelopment. However, there are several single cell / nuclei RNA-Seq datasets from the human prenatal brain available, which include neural progenitor cells (cycling progenitors / radial glia) similar to NSCs as well as developing glutamatergic and GABAergic neurons, deriving from regions such frontal cortex and hippocampus (e.g. PMID: 36150908), which the authors could additionally use for these analyses.

***Response 1.6***

*Thank you for this helpful suggestion and it mirrors a suggestion by Reviewer 2 (#2.6), i.e., to consider using human single cell data. We have now done this by testing our gene-set for enrichment in two human data-sets; the prenatal dataset (Cameron et al, 2023) suggested here and an adult dataset (Hodge et al, 2019, Tasic et al, 2018). We have updated the Methods and Results sections to include these analyses. Overall, the three gene-sets with by far the most enriched cell types are the three gene-sets that were enriched for common variation associated with SCZ, IQ and EA and rare DNMs reported in neurodevelopmental disorders (NSCs_DEL^hom^, iNs_DEL^het^_PB and iNs_DEL^hom^_PB). There is a consistent pattern for these gene-sets in the prenatal and adult data with both glutamatergic excitatory neurons and GABAergic inhibitory neurons enriched across different regions of the prenatal brain and across regions of the adult cortex. In addition, the NSCs_DEL^hom^ gene-set was enriched within cycling progenitor cells (as noted by the Reviewer above) and intermediate progenitor cells within the prenatal brain, which like the NSCs can produce new types of neurons and glial cells.*

*We had included the following as a limitation of this study: “In addition, the single-cell RNA seq dataset employed in this study does not contain data from a stage of development equivalent to NSCs. Therefore, while we could investigate which cell types in the adult mouse were enriched for genes in the NSCs set, we were not able to investigate that in a single cell dataset from an early stage of neurodevelopment.” We have now deleted it from the Discussion.*

**Reviewer #2:**

This study aims to investigate if the direct targets of MEF2C are enriched for genes containing common GWAS genetic variants associated with SCZ and cognitive function, as well as enriched for genes harboring rare de novo mutations (DNMs) contributing to neurodevelopmental disorders. Then, biological processes and specific cell types that are dysregulated due to MEF2C disruption are investigated, and finally, the authors looked into the trans-eQTLs at the MEF2C gene that are associated with altered expression of MEF2C target genes. Transcriptmics data and differentially expressed genes were previously generated by another group. Chip-seq data human fetal brain cultures were generated in this study.

Overall assessment:

the study is interesting, and the findings are relevant for describing the putative molecular mechanisms of the MEF2C gene in schizophrenia and cognitive dysfunction. However, some of the conclusions are only weakly supported by the results presented.

Below, I provide detailed comments for the authors to address, and I also suggest ways to strengthen the results and further substantiate the main conclusions.

**Major points**

**(2.1)** The Chip-Seq data should be analyzed and reported in greater detail. The authors should report the number of peaks, genes and functional enrichment results for the genes with an overlapping Chip-Seq signal. This will help interpreting the enrichment results that are presented later in the manuscript.

***Response 2.1***

*Firstly, to clarify, we stated in the original submission that “we utilized existing ChIP-seq data from a study of MEF2C conducted on human fetal brain cultures [18].” We did not generate the ChIP-seq data ourselves. Like the RNA-seq data, we sourced it from a previous publication. However, we are happy to perform the additional analyses requested here. We have updated the Results to now start with a section entitled “Identification and Annotation of MEF2C Binding Peaks” that describes the number of ChIP-seq peaks found and their proximity to gene-encoding regions (new S6 Table). We could map these peaks to 5,775 protein coding genes and performed functional enrichment GO analyses of these, which showed these genes were most involved in RNA binding, transcription and functions within the nucleus, as summarized in Results and detailed in a new S7 Table. This is as expected given MEF2C’s role as a transcription factor. Importantly, the GO terms that emerge later when we analyze subsets of genes that have been dysregulated following KO of MEF2C (e.g. related to neuron development and differentiation, and oxidative phosphorylation and mitochondrial function) were not initially highly enriched for these 5,775 protein coding genes. Those GO terms only emerge after the function of MEF2C has been disrupted.*

*We understand that this is the point that the Reviewer is making here when stating “This will help interpreting the enrichment results that are presented later in the manuscript”. The enriched GO terms following KO of MEF2C are distinct from those observed from the ChIP-seq binding pattern of MEF2C in the absence of any gene disruption. We have now noted this in Results.*

**(2.2)** It will strengthen the manuscript if the Authors validate (e.g., by Chip qPCR) some of the most relevant targets of MEF2C identified in Table 1 that are associated with genetic signals for SCZ, IQ, and EA.

***Response 2.2:***

*We agree with this comment but as highlighted above, we did not generate these data ourselves and cannot validate some results, e.g., by ChIP qPCR.* *We have now updated the Discussion to acknowledge that not validating MEF2C binding at the most relevant targets of MEF2C represents a limitation of this study. However, the new functional enrichment analysis, helpfully suggested above, does identify that genes in proximity to MEF2C ChIP-seq peaks are involved in biological processes such as transcription and translation that are known to be regulated by MEF2C.*

*In our study, we utilized a ranking-based method to identify the most relevant target genes by integrating RNA-seq data with ChIP-seq data using BETA package software. This allowed us to analyze genes that are both differentially expressed and have a regulatory potential. Each gene is assigned two ranks: One rank is based on the regulatory potential score, which is calculated by evaluating the number of binding sites and their proximity to the transcription start site. The other rank is based on the FDR, with the most strongly differentially expressed gene given the top rank. The rank product (RP) for each gene is then calculated by combining these two ranks, reflecting both the regulatory potential and the differential expression. The RP value can be interpreted as a probability, indicating the likelihood that a gene is a true target based on both criteria. We set a conservative threshold such that genes with an RP below a certain cutoff (probability less than 0.01) are considered more likely to be true target genes. By employing this method, we aimed to identify and prioritize genes that are both strongly differentially expressed and have high regulatory potential. We have updated the Methods to highlight this point.*

**(2.3)** GWAS enrichment analysis: what is the overlap between SCZ, IQ, and EA GWAS signals? To assess specificity of the GWAS enrichments, the authors should first identify disease-specific GWAS gene sets, and similarly test the 8 DEGs for heritability enrichment.

*See 2.4 below.*

**(2.4)** Related to the previous point, the presence a (potential) substantial overlap of GWAS signals between SCZ, IQ, and EA, might explain why the three gene-sets enriched for common variation associated with SCZ, IQ and EA also explain a significant proportion of variance in IQ. The authors should rule out this scenario before affirming that genetic variants within these gene-sets play a role in influencing both susceptibility to SCZ and cognitive performance. I suggest looking for a potential overlap of GWAS signals between SCZ, IQ, and EA, identify gene sets specific for each disease, and take these shared genes and disease-specific genes into account in their analyses.

***Responses 2.3 and 2.4***

*We will respond to these two related points together. To address this issue we first calculated the genetic correlation between SCZ, IQ and EA using LDSC and GWAS data (data not shown but consistent with previous reports of the same analysis). As expected, there was a highly significant correlation between IQ and EA. SCZ is correlated with IQ but not with EA. To further explore the shared genetics between these phenotypes, we then listed the genes reported as associated in each of the original GWAS. That is 682 genes associated with SCZ, 1,016 genes associated with IQ and 1,838 genes associated with EA. Given the strong genetic correlation between the two cognitive phenotypes, we collapsed these data into three lists of genes; genes that are associated with SCZ but not the cognitive phenotypes (IQ or EA; n=472), genes that are associated with at least one of the cognitive phenotypes (IQ or EA) but not SCZ (n=2,258) and genes that are associated with both SCZ and at least one of the cognitive phenotypes (IQ or EA; n=210).*

*Using these data, we could then generate versions of our 8 gene-sets that do not include phenotype-specific associated genes and test for heritability enrichment as suggested. For example, we could take a gene-set that is enriched for heritability for SCZ, IQ and EA and (i) remove all genes associated with SCZ (including SCZ-associated genes that are also associated with IQ or EA as well) and test if it is still enriched for IQ or EA, and (ii) remove all genes associated with IQ or EA (including IQ- or EA-associated genes that are also associated with SCZ as well) and test if it is still enriched for SCZ.*

*When we removed genes associated with SCZ from the enrichment analysis of IQ and EA, we saw that the majority of enriched gene-sets remained significant. When we removed genes associated with IQ or EA from the enrichment analysis of SCZ, we saw that only the enrichment of the NSCs_DEL^hom^ gene-set remained significant. This suggests that some MEF2C target genes are contributing to both SCZ and cognitive phenotypes while others are more phenotype specific. We have added text to the Results subsection entitled “Enrichment Analysis for Genes Containing Common Variants” that details this and we have added a new S8B Table showing these data.*

*We also used these data on genes associated with different phenotypes to perform similar follow-up analysis of the PRS analysis. When we removed genes associated with IQ or EA from the MEF2C target gene-sets, the three gene-set-based SCZ-PRSs could still explain variance in IQ in an independent dataset at levels that were nominally significant, indicating that genetic variants within these gene-sets play a role in influencing both susceptibility to SCZ and cognitive performance* *and the effect was not just due to genes associated with IQ or EA. We have added text to the Results subsection entitled “Genetic Association between SCZ and Cognitive Function” that details this and we have added to S10B Table showing these data.*

**(2.5)** The authors should assess the stability of the results presented in Figure 3 by performing a sensitivity analysis with respect to the p-value threshold for SNP inclusion. This will strengthen the conclusions of the gene-set based PRS analysis.

***Response 2.5***

*We had stated in Methods that “Effect-size weighted SCZ-PRS and IQ-PRS were computed for each gene-set using thresholds ranging from P < 0.05 to P<1” but we had not specified the thresholds used, nor had we commented on the stability of the results. We have now updated the Methods to detail the different thresholds used and we have updated the Results subsection entitled “Genetic Association between SCZ and Cognitive Function” to indicate that we performed a sensitivity analysis with respect to the p-value threshold for SNP inclusion and found that results were consistent and stable across different p-value thresholds, with these data displayed in S10A Table.*

**(2.6)** “We used a single-cell RNA sequencing (scRNA-seq) dataset with data from 565 cell types across nine brain regions of the mouse brain [40].” What is the rationale for using mouse data instead of human data for this analysis? Are there human single cell data that can be used for this enrichment analysis? E.g., <https://celltypes.brain-map.org/> or data reported by Siletti et al., Transcriptomic diversity of cell types across the adult human brain. Science 382, eadd7046 (2023). DOI: 10.1126/science.add7046? Can cross-species differences affect the enrichments results?

***Response 2.6***

*This is a good suggestion and has been addressed in response to Reviewer 1 above (#1.6) where we replace the mouse-based single cell analysis with cell-type enrichment analyses using both prenatal and adult human datasets.*

**(2.7)** The background gene-set used in the functional enrichment analysis (by ClueGO) was “brain tissue-expressed genes”. Given the cell-type specific enrichments (Table 3), I suggest repeating this analysis using cell-type expressed genes. This might provide better insights into the specificity of the functional enrichments results presented in the Functional Enrichment Analysis section. I also suggest expanding this analysis beyond GO terms, and explore pathways (KEGG, Panther, Biocarta, etc) which provide information about more specific cellular processes.

***Responses 2.7***

*We agree with these suggestions. We have now expanded the functional enrichment analysis to explore additional biological pathways (KEGG, Reactome, WikiPathways). For the MEF2C direct target gene-sets, we now have used cell-type specific expressed genes as the background gene-set. These have been derived using the raw reads counts from the RNA-seq data for the wild type (control) NSCs and iNs from the original data. The results remain largely consistent with what we reported previously. Investigating the additional biological pathways (e.g. KEGG) produced consistent results with those from the GO analysis (enrichment of pathways related to mitochondrial function) but does highlight some additional pathways, e.g. Orexin receptor pathway.*

**(2.8)** Furthermore, I suggest the authors to (1) present graphs with the degree of enrichment (e.g., significance, enrichment score, etc) rather than the pie charts in Supplementary Figures 2-5 and Supplementary Figures 6-9, and (2) summarise the main (key) results of the Functional Enrichment Analysis in a main figure.

***Responses 2.8***

*We agree with this suggestion. We have now dropped the pie charts from the supplementary figures and generated new Figures 4-6 that displays a summary of the functional enrichment analysis.*

**(2.9)** Trans Expression Quantitative Trait Loci Analysis. “For the five gene-sets that were enriched for association with at least one of SCZ, IQ or EA, we overlapped the MEF2C direct target genes in the enriched GO terms (Supplementary Table 15) with the 120 genes prioritized in the latest GWAS for SCZ (Supplementary Table 16) [7].” The Authors should provide a clear justification for this analysis. For example, why focusing on the latest GWAS for SCZ? Similarly, why the MEF2C direct target genes in the enriched GO terms? What is the aim of this analysis? Adding these details and explaining the rationale and hypothesis tested by this analysis will help the reader to understand the results.

***Response 2.9***

*The aim of the Trans Expression Quantitative Trait Loci Analysis was to investigate if any of the 10 genome-wide significant LD-independent SNPs at MEF2C that were associated with SCZ, EA and IQ were also associated with altered expression of a downstream MEF2C target gene. If MEF2C is regulating the expression of these genes, we hypothesized that genetic variation at MEF2C (associated with SCZ, EA or IQ) could indirectly affect expression of a downstream target gene, mediated through MEF2C’s role as a transcription factor. This would be a trans eQTL effect and evidence of two risk genes (i.e., MEF2C and a downstream target gene) functioning within a putative risk pathway.*

*We could test 10 SNPs at MEF2C in 13 brain tissue types from GTEx against each MEF2C direct target gene. As there are thousands of MEF2C direct target genes, this multiplies up to a major multiple testing problem. The challenge here is that the statistical power of the GTEx dataset (source of eQTL data) is limited due to small sample size numbers. We wanted to reduce the number of tests by reducing the number of MEF2C direct target genes to be analyzed. Our first filter was to restrict the analysis to MEF2C direct target genes that are associated with SCZ, IQ or EA. As detailed earlier, that still represents hundreds of genes. The SCZ GWAS study performed an in-depth gene prioritization analysis (unlike the GWASs of the IQ and EA) using fine-mapping and functional genomic data. This identified just 120 genes that are likely to underpin associations at the reported loci. To reduce this number further, we reasoned that if the GO analysis suggested different biological processes that are enriched for MEF2C direct target genes, we should use this to guide us in selecting a smaller number of genes for this analysis. Hence, the MEF2C direct target genes selected were those from the 120 SCZ high confidence genes that were in the enriched GO terms.*

*We agree that we had not stated our hypothesis here and provided detail on our rationale for the approach used. We have updated the Trans Expression Quantitative Trait Loci subsection of the Results to now include this information.*

**(2.10)** The identification of BNIP3L as a trans regulated gene by a SNP at MEF2C locus is of interest, but on its own this is not sufficient to support a “role of MEF2C and mitochondrial dysfunction in the etiology of the studied disorders”. Also, as pointed out by the authors, since “approximately 28% of the eQTL genes implicated in SCZ were related to mitochondria [58]”, the authors should provide more convincing evidence to strengthen the role of MEF2C in mitochondrial dysfunction. For example, I would suggest this analysis to be expanded to test the underlying hypothesis more comprehensively: e.g., are the genes from the iNs gene-sets enriched for GO terms related to mitochondrial function and energy production more likely to be regulated in trans by MEF2C? Perhaps this eQTL analysis could even be refined by accounting for genes with cell-type specific expression (enrichments presented in Table 3) to improve sensitivity.

***Response 2.10***

*We had stated that “These findings provide further support for the potential role of MEF2C and mitochondrial dysfunction in the etiology of the studied disorders.” We have now removed this sentence as we agree that the trans eQTL analysis alone is not sufficient support for this. We did perform an additional analysis and expanded the trans eQTL analysis as suggested by the Reviewer above. We restricted the target genes to only those in enriched GO terms related to mitochondrial function and energy production and to only those with cell-type specific expression in the enriched cell-types from that earlier analysis. As a result, we tested the 10 LD-independent SNPs at MEF2C against 300 genes. Overall, we found that none of the tested SNPs showed a trans eQTL effect on the expression of any gene from the 300 genes tested except for BNIP3L, which we had detected already.*

**Minor comments**

**(2.11)** “SNP P values and effect sizes for SCZ were derived from a SCZ GWAS meta-analysis on 183 40,675 cases and 64,643 controls [7] (excluding Irish samples)”. What is the rationale to exclude Irish samples?

***Response 2.11***

*Irish samples were excluded from this GWAS to keep that base/discovery sample independent from the target/test sample for the PRS analysis. If not independent, it can confound the analysis. We have updated the Methods to clarify this.*

**(2.12)** “We used a single-cell RNA sequencing (scRNA-seq) dataset with data from 565 cell types across nine brain regions of the mouse brain [40].” What is the rationale for using mouse data instead of human data for this analysis? Can cross-species differences affect the enrichments results?

***Response 2.12***

*See response to 1.6 and 2.6 above.*

**(2.13)** Table 1. it will be helpful to report percentages (%) as well as absolute numbers.

***Response 2.13***

*Percentages have now been added to the last column of that table.*
