# Supplementary material for: Direct targets of MEF2C are enriched for genes associated with schizophrenia and cognitive function and are involved in neuron development and mitochondrial function
Source: PLoS Genet. 2024 Sep 11;20(9):e1011093. doi: 10.1371/journal.pgen.1011093 (PMC11419381; doi:10.1371/journal.pgen.1011093)
Supplement: Attachment — Submitted filename: Responses to Reviewers Comments 2.docx [file pgen.1011093.s031.docx]

**Comments to the Authors:**

Reviewer #1: The authors have addressed my comments, with interesting additional results.

I have 2 suggestions for minor amendment of the text:

1.1: Lines 176 - 177: 'Enrichment for heritability was compared to the baseline model using a corresponding P value
(one-tailed to identify significant local SNP-heritability).'

I think it would be clearer to state: 'Enrichment of SNP heritability was compared to the baseline model, using the Z-score to derive a (one-tailed) P-value.'

*Response 1.1: This change has been made.*

1.2: Line 354: 'Although these sets of DEGs have been generated in differentiated neuronal cell types,...'

Aren't some DEGs taken from neural stem cells? If so, I recommend deleting this part of the sentence.

*Response 1.2: This change has been made.*

Reviewer #3: The primary aim of this research is to investigate direct target of MEFC2 which could play an important role in SCZ risk and the regulation of cognitive function. The study integrates RNA-seq data from iPSCs-derived NSCs and induced neurons, in which MEF2C function is disrupted with CHIP-seq data of MEF2C. It is a well-organized work that progressively identified that several MEF2C direct target gene-sets are enriched for SNP-based heritability for cognitive function and SCZ, as well genes containing rare de novo mutations reported in diseases studied. The specific involved biological processes and trans expression quantitative trait locus effect of a single SNP at MEF2C on the expression of a target gene, BNIP3L are also interestingly observed. This article is logical, informative and uses analyses skillfully. Suggestions for improvement of the manuscript are followed.

3.1: Abstract: The authors state that ‘we investigated the involvement of MEF2C in these 22 phenotypes using human-derived neural stem cells (NSCs) and induced neurons (iNs)’, but in the research, only glutamatergic neurons are involved. There could be more precise formulation. Or else, incorporating other types of neurons into the study might be better.

*Response 3.1: The reviewer is correct in that only data from glutamatergic induced neurons was available for this analysis. We have updated the Abstract to clarify this.*

3.2: Figure 1: The CHIP-seq data used in this research is from human fetal brain tissue, what’s the reason for choice or, why not choose an adult one?

*Response 3.2: The only CHIP-seq data that we could access was from human fetal brain tissue, with none available from adult human brain tissue. This fetal brain tissue-based data is well suited to analysis of the RNA-seq data from the neural stem cells whereas adult brain tissue-based data would have been better for induced neurons. Ultimately, ChIP-seq and RNA-seq data from the same samples would have been best. We do acknowledge that in the limitations at the end of the Discussion when saying that “A first limitation of this study is that the ChIP-seq data was not generated from the same human neural cell models as the RNA-seq data, it came from human fetal brain cultures. Ideally, these data would come from the same source when trying to combine them to identify direct target genes.”*

3.3: More convincing evidence should be provided to make robust link of MEF2C with mitochondrial dysfunction.

*Response 3.3: We are not in a position to provide more evidence based on the data generated for this study. However, we have added additional information to the Discussion of this topic from a recent study that showed that mutation of MEF2C leads to neuronal mitochondria dysfunction and motor deficits in mice.*

3.4: Page 21: It may be abrupt if BNIP3L is pointed out directly, I suggest the description like ‘none of the tested SNPs showed a trans eQTL effect on the expression of any gene except for the finding for BNIP3L already detected’ could be arranged forward.

*Response 3.4: We agree and have changed the text accordingly.*
